# Self-synergizing mutual prodrug liposomes for targeted cancer therapy *via* redox-amplified Pin1 inhibition

**DOI:** 10.7150/thno.131597

**Published:** 2026-06-10

**Authors:** Nuri Kim, Ilseob Kim, Hanui Jo, Nanhee Song, Sangmin Jo, Suyeon Lee, Hoechang Kim, Changjin Lim, Dongwon Lee

**Affiliations:** 1 Department of Bionanotechnology and Bioconvergence Engineering, Jeonbuk National University, Jeonju, Jeonbuk, 54896, Republic of Korea.; 2 Department of Pharmacy, Jeonbuk National University, Jeonju, Jeonbuk, 54896, Republic of Korea.; 3 Department of Polymer• Nano Science and Technology, Jeonbuk National University, Jeonju, Jeonbuk, 54896, Republic of Korea.

**Keywords:** all-trans retinoic acid, prodrug, cancer, liposome, Pin1, redox homeostasis

## Abstract

**Rationale:**

Combination chemotherapy often suffers from poor pharmacokinetics, asynchronous drug delivery, and limited synergism. We anticipated that combining all-*trans* retinoic acid (atRA) with a quinone methide (QM) precursor would promote intracellular reactive oxygen species (ROS) accumulation through distinct yet complementary mechanisms. We further hypothesized that this redox disruption could ultimately suppress the oncogenic protein Pin1, resulting in synergistic anticancer effects.

**Method:**

We designed proxiRQ, a mutual prodrug in which atRA and a QM precursor are linked *via* an esterase-cleavable bond. Owing to the amphiphilic nature of proxiRQ, it was able to self-assemble with dipalmitoyl phosphatidylcholine (DPPC) to form cholesterol-free liposomes. We obtained tL-proxiRQ by further surface modification with poly(γ-glutamic acid) (γPGA), which improved systemic circulation and enhanced tumor accumulation. Subsequently, we evaluated physicochemical properties, esterase-triggered drug release, cellular redox modulation, and* in vitro* and *in vivo* antitumor efficacies.

**Results:**

tL-proxiRQ, featuring a high drug loading capacity (37 mol%) and colloidal stability, synchronously released atRA and QM upon esterase activation in tumor cells. QM-mediated glutathione (GSH) depletion amplified atRA-induced oxidative stress, thereby enhancing Pin1 expression inhibition. Compared to the free drug combination, tL-proxiRQ demonstrated significantly enhanced cytotoxicity, greater downregulation of Pin1, and enhanced apoptosis induction. *In vivo*, tL-proxiRQ achieved potent tumor growth inhibition in the MCF-7 xenograft model with minimal systemic toxicity.

**Conclusion:**

This study validates proxiRQ as a self-synergizing mutual prodrug and introduces tL-proxiRQ as a rationally engineered nanoplatform that integrates mutual prodrug chemistry, synergistic redox modulation, and targeted liposomal delivery to overcome the key limitations of conventional combination therapy.

## Introduction

atRA is an active derivative of retinol and a member of the vitamin A family and plays an essential role in regulating cell growth, proliferation and apoptosis. atRA has been successfully used as a therapeutic agent for acute promyelocytic leukemia owing to its ability to induce cell differentiation and apoptosis while suppressing tumor cell proliferation 1-3. In addition, atRA is known to modulate ROS levels, contributing to its anticancer activity. While basal levels of ROS are essential for normal cellular signaling and redox homeostasis, excessive ROS accumulation leads to oxidative stress and cancer cell death. This ROS-mediated mechanism enhances the therapeutic efficacy of atRA and increases the sensitivity of cancer cells to conventional anticancer therapy 4, 5. Beyond its role in ROS regulation, atRA inhibits the prolyl isomerase Pin1 (peptidyl-prolyl *cis-trans* isomerase NIMA-interacting 1), an enzyme that catalyzes *cis-trans* isomerization of phosphorylated Ser/Thr-Pro motifs 6. Pin1 is overexpressed in most cancer cells and acts as a crucial regulator of several cancer-associated pathways by stabilizing oncogenic proteins, interfering with the tumor suppressor p53, and promoting metastasis 7. Notably, atRA inhibits Pin1 through both direct ligand binding 8, 9 and indirect oxidative modification of its Cys113 residue. Since oxidized Pin1 is particularly vulnerable to redox-mediated inactivation under oxidative stress, an excess accumulation of ROS can exert a synergistic effect with atRA, leading to potent Pin1 inhibition and enhanced anticancer activity 10.

Cancer cells are characterized by an altered redox balance, which manifests as elevated ROS levels and the activation of antioxidant defense systems. Among intracellular antioxidants, GSH is the most abundant and plays a key role in maintaining redox homeostasis and supporting cancer cell survival under oxidative stress. Therefore, selective GSH depletion further disrupts the fragile redox balance of cancer cells, sensitizing them to ROS-mediated therapy without affecting normal cells 11-13. Based on this principle, various GSH-depleting agents have been studied as oxidative stress-amplifying anticancer strategies 14, 15. QM is a highly electrophilic intermediate known to possess strong GSH-scavenging ability. QM can be generated *via* the spontaneous 1,6-elimination of phenolate from acetate-derived esters or aryl boronic esters containing ortho-substituents with a leaving group 16, 17. Accumulating evidence indicates that QM effectively depletes GSH and amplifies oxidative stress, leading to selective cancer cell death 18, 19. Based on these complementary mechanisms, we designed proxiRQ, a novel self-synergizing mutual prodrug that incorporates atRA and a QM precursor into a single molecular scaffold. This design enables the simultaneous release of both active agents upon esterase-mediated cleavage. Upon esterase-mediated intracellular activation, atRA induces oxidative stress, binds directly to and inhibits Pin1, and promotes apoptosis 9, 20. Concurrently, the released QM depletes intracellular GSH, weakening antioxidant defenses and promoting the accumulation of excess ROS, thereby further enhancing redox-mediated Pin1 inactivation 17. This intramolecularly coordinated design differentiates proxiRQ from existing combination therapies by enabling it to regulate its own synergistic anticancer actions through interconnected redox pathways.

Although atRA has a strong biological rationale for cancer, particularly estrogen receptor-positive breast cancer, through Pin1 inhibition and RARα (retinoic acid receptor alpha) modulation, its clinical application in solid tumors has been severely limited due to its rapid systemic clearance and low intratumoral accumulation 21-23. To overcome these pharmacokinetic limitations and address delivery issues associated with small-molecule drugs, liposomes have been widely used as carrier systems due to their biocompatibility and ability to encapsulate both hydrophobic and hydrophilic drugs 24. In conventional liposomal formulations, hydrophobic drugs are inserted into the lipid bilayer *via* hydrophobic interactions. However, such systems often suffer from low drug loading efficiency (typically <10 wt%), limited storage stability and premature drug leakage 25-28. Drug molecules with linear geometry and distinct hydrophobicity are more likely to align with lipid acyl chains and can be stably inserted into the lipid bilayer at high loading levels. Structurally, proxiRQ is an amphiphilic hybrid prodrug characterized by a rigid hydrophobic polyene tail and a polar carboxylic acid head group. Therefore, we hypothesized that proxiRQ could potentially replace cholesterol by stably incorporating into lipid bilayers, functioning not only as a therapeutic agent but also as a membrane-stabilizing component.

Accordingly, the liposome (L-proxiRQ) was prepared by co-formulating proxiRQ with DPPC in a 4:6 molar ratio, thereby achieving a drug content of 37%, far exceeding that of conventional liposomes, while maintaining physicochemical stability. To further enhance tumor selectivity and improve pharmacokinetic performance, L-proxiRQ was surface-modified with γPGA, which targets γ-glutamyl transferase (GGT), an enzyme overexpressed on the membrane of various cancer cells 29-31. The resulting γPGA-coated proxiRQ-loaded liposomal formulation (tL-proxiRQ) integrates efficient drug loading, bilayer stabilization and enzyme-mediated tumor targeting into a single nanoplatform.

In this study, we systematically examined the physicochemical properties, drug release profiles, and anticancer efficacy of tL-proxiRQ in both cell culture and mouse xenograft models. The results of this study demonstrate that proxiRQ-based liposomes enable the synchronized delivery of atRA and QM, utilizing complementary redox-modulating mechanisms: QM depletes GSH and weakens antioxidant defenses, thereby amplifying atRA-induced intracellular ROS accumulation and ultimately enhancing Pin1 inhibition. This intermolecular cooperation underpins the synergistic anticancer activity of tL-proxiRQ and establishes self-synergizing mutual prodrug liposomes as a promising platform for redox-mediated, Pin1-targeted and GGT-directed cancer therapies.

## Materials and Methods

### Materials

Commercially available reagents and solvents were used without additional purification unless specified. Tetrahydrofuran (THF) was distilled from sodium benzophenone, and dichloromethane (DCM) was freshly distilled from calcium hydride. All solvents used for product isolation and chromatography were reagent-grade and glass-distilled. Flash column chromatography was performed on silica gel 60 (230–400 mesh, Merck) with the indicated solvents.

### Synthesis and characterization of proxiRQ

4-Hydroxybenzyl alcohol (HBA, 3.3 mmol) was first dissolved in 3.5 mL of dimethylformamide (DMF), and K_2_CO_3_ (5.0 mmol) was added at room temperature. After stirring the solution for 30 min, allyl bromide (5.0 mmol) was carefully added dropwise. The reaction was allowed to proceed overnight at room temperature. Upon completion, the mixture was quenched with aqueous HCl and extracted with diethyl ether. The combined organic layer was washed with brine, dried over anhydrous MgSO_4_, and evaporated under reduced pressure. The residue was purified by flash column chromatography, yielding compound** 1** (4-(allyloxy)phenyl)methanol as a yellow oil.

To synthesize compound **3**, a multistep sequence was employed. Initially, compound** 2** (4-oxo-4-(2-(trimethylsilyl)ethoxy)butanoic acid) was prepared by suspending succinic acid (2.2 mmol) in toluene (4.0 mL), followed by the addition of 2-(trimethylsilyl)ethanol (2.0 mmol), *N*,*N*-diisopropylethylamine (DIPEA, 1.1 mmol), and 4-(dimethylamino)pyridine (DMAP, 0.22 mmol) at room temperature. The mixture was stirred overnight at 80 °C, quenched with NH_4_Cl, and extracted with ethyl acetate (EtOAc). The organic layer was dried and concentrated, and the residue was purified by flash column chromatography to afford compound **2** as a yellow oil. In the next step, compound **2′** (4-(allyloxy)benzyl (2-(trimethylsilyl)ethyl) succinate) was synthesized by reacting compound **2** (1.7 mmol) with compound **1** (2.0 mmol), DMAP (0.33 mmol), and EDC·HCl (3.3 mmol) in CH_2_Cl_2_ (8.0 mL) at room temperature. After stirring overnight, the mixture was extracted with CH_2_Cl_2_ and purified by flash column chromatography to yield compound **2′**. Compound **3** was subsequently prepared by dissolving compound **2′** (1.6 mmol) in THF (6.0 mL), followed by the addition of *N*-methyl aniline (8.0 mmol) and Pd(PPh_3_)_4_ (0.32 mmol) at room temperature. The reaction mixture was stirred for 2 h, extracted with EtOAc, and purified by flash column chromatography to afford compound **3** as a yellow oil.

Compound **5** was synthesized in the following manner. atRA (4.4 mmol) was dissolved in CH_2_Cl_2_ (8.0 mL), and Et_3_N (13 mmol) and pivaloyl chloride (4.4 mmol) were added at room temperature. After stirring for 30 min, DMAP (0.59 mmol) and compound **3** (1.5 mmol), previously dissolved in CH_2_Cl_2_ (2.0 mL), were added. The reaction was stirred overnight, extracted with CH_2_Cl_2_, and the organic layer was purified by flash column chromatography to afford compound **5** as a yellow oil.

Finally, proxiRQ was synthesized from compound **5**. Compound **5** (0.69 mmol) was dissolved in THF (3.0 mL) and cooled to 0 °C. Tetra-*n*-butylammonium fluoride (1.0 M in THF, 1.0 mmol) was added, and the mixture was stirred at 0 °C for 6 h. The reaction was then quenched with saturated NH_4_Cl and extracted with EtOAc. The organic layer was washed with brine, dried, and concentrated under reduced pressure. The residue was purified by flash column chromatography to yield proxiRQ as a yellow solid (57% yield). The structure of proxiRQ was confirmed by 500 MHz ^1^H NMR spectrometer (JNM-ECZ500R, JEOL, Japan) using CDCl_3_. To evaluate structural and molecular weight changes in the presence or absence of esterase at 37 °C, proxiRQ was treated with an esterase solution for predetermined time, followed by lyophilization. The obtained samples were analyzed using ^1^H NMR and LC-MS/MS (6410 Triple Quad LC/MS, Agilent Technologies, Santa Clara, CA, USA). Furthermore, the GSH scavenging ability was evaluated using Ellman’s reagent (5,5′-dithiobis (2-nitrobenzoic acid)) (Sigma-Aldrich, St Louis, MO, USA) after incubating proxiRQ with the esterase solution. The Pin1 inhibitory activity of proxiRQ was measured using the SensoLyte^®^ Green PIN1 Activity Assay Kit (AnaSpec, CA, USA). Briefly, the Pin1 enzyme solution and test compounds were incubated for approximately 1 h, and the reference inhibitor provided in the kit was used as a positive control.

### Preparation of L-proxiRQ and tL-proxiRQ

L-proxiRQ was prepared using proxiRQ and amphiphilic lipid DPPC by the reverse-phase evaporation method. proxiRQ and DPPC were dissolved in chloroform at a molar ratio of 4:6 to obtain a homogeneous lipid solution. The solution was transferred to a 500 mL round-bottom flask with an extended neck, and the chloroform was removed under vacuum using rotary evaporator, forming a thin lipid film on the inner walls of the flask. The lipid film was further dried under vacuum pressure for 24 h to completely remove the solvent. The dried lipid film was hydrated using a two-phase system consisting of diethyl ether and distilled water. First, diethyl ether (8.0 mL) was added to the round-bottom flask to facilitate lipid film detachment from the flask walls, thereby promoting uniform hydration. Then, distilled water (8.0 mL) was added under a nitrogen atmosphere to promote liposome formation. The mixture was sonicated for 3 min and then vortex-mixed for uniform dispersion and liposome formation. Subsequently, diethyl ether was removed under high vacuum to obtain L-proxiRQ. tL-proxiRQ was prepared by coating γPGA on the surface of L-proxiRQ through electrostatic interactions. Specifically, γPGA (15 wt%) dissolved in distilled water was added dropwise to 8.0 mL of L-proxiRQ (1 mg/mL) and stirred at room temperature for 10 min. To isolate tL-proxiRQ, the mixture was ultracentrifuged at 50,000 × *g* for 1 h at 4 °C. Finally, the concentration of tL-proxiRQ was roughly adjusted using phosphate-buffered saline (PBS).

### Characterization of L-proxiRQ and tL-proxiRQ

The morphology and size of L-proxiRQ and tL-proxiRQ were characterized using transmission electron microscopy (TEM; H-7650, HITACHI, Tokyo, Japan), cryogenic transmission electron microscopy (cryo TEM; Talos, L120C, Thermo Scientific, Waltham, MA, USA), and a particle size analyzer (90 Plus, Brookhaven Instruments Corp., Holtsville, NY, USA), respectively. The zeta potential distribution of L-proxiRQ and tL-proxiRQ was measured using a zeta potential analyzer (ELSZ-2000 series, Otsuka Electronics, Osaka, Japan). To assess colloidal stability, the size of the tL-proxiRQ dispersed in pH 7.4 PBS, and PBS containing fetal bovine serum (FBS; Gibco), urea, and NaCl was analyzed using a particle size analyzer.

The drug loading capacity (DLC) of tL-proxiRQ was determined using ^1^H NMR spectroscopy. Briefly, the purified tL-proxiRQ dispersion was lyophilized, and the resulting solid was dissolved in CDCl_3_. After removing insoluble inorganic salts *via* centrifugation, the supernatant was analyzed. DLC was calculated as the molar percentage (mol%) of proxiRQ relative to the total lipid content (proxiRQ + DPPC), based on the integration ratio of the benzyl protons of proxiRQ to the N-methyl protons of DPPC. To evaluate the estimated encapsulation efficiency (EE), the accurate stoichiometric ratio obtained from the ^1^H NMR data was used. Assuming that DPPC loss was minimized during the ultracentrifugation process, the estimated EE was calculated as the ratio of the experimentally measured moles of proxiRQ contained in the final liposome pellet to the initial moles of proxiRQ used for formulation.

### Cumulative drug release

The cumulative release of QM from tL-proxiRQ (1.0 mg/mL) was assessed using a dialysis method. The liposomal formulation was placed inside a dialysis membrane with a molecular weight cut-off of 15 kDa and immersed in PBS (pH 7.4) at 37 °C. Drug release was assessed under two conditions: in the absence and presence of esterase. At predetermined time points, 2.0 mL of the external medium was collected and replaced with an equal volume of fresh PBS. Because of the high reactivity of QM in aqueous environments, HBA was used as a surrogate marker to quantify QM release. The concentration of HBA released from the collected samples was quantified using a UV-vis spectrophotometer (Scinco, Seoul, Republic of Korea) based on a standard calibration curve. The percentage of cumulative release was calculated based on the maximum amount of QM theoretically encapsulated within the liposomes.

### Cell culture

The human breast cancer cell line (MCF-7), murine macrophage cell line (RAW 264.7), mouse renal tubular epithelial cell line (TCMK-1), and human embryonic kidney cell line (HEK-293) were obtained from the Korea Cell Line Bank (Seoul, Republic of Korea). The cells were maintained in Dulbecco’s Modified Eagle’s Medium (DMEM) supplemented with 10% FBS and 1.0% antibiotic-antimycotic (Gibco). All cell cultures were maintained in a humidified incubator at 37 °C with 5% CO_2_.

### Cellular uptake and endosomal escape of tL-proxiRQ

To evaluate the cellular uptake and endosomal escape of tL-proxiRQ, MCF-7 cells were seeded in a glass-bottom dish at a density of 4.0 × 10^4^ cells per dish. After overnight incubation, the cells were treated with Nile Red-loaded tL-proxiRQ (75 µg/mL) for 0.5, 1, 3, and 6 h. Following incubation, the cells were washed with PBS and subsequently stained with LysoTracker Green (Invitrogen, Waltham, MA, USA) to visualize endo/lysosomal compartments. For baseline comparison, cells stained only with LysoTracker (without tL-proxiRQ treatment) were used as the control to visualize the normal lysosomal distribution. After an additional PBS wash, fluorescence images were acquired using a confocal laser scanning microscope (LSM 880 with Airyscan Carl Zeiss, Germany). Co-localization between Nile Red-loaded tL-proxiRQ and LysoTracker Green was quantitatively analyzed using ImageJ software to assess endosomal localization and escape behavior.

To further investigate the role of GGT in tL-proxiRQ internalization, MCF-7 cells were pretreated with either a GGT inhibitor (GGsTop, MedchemExpress, NJ, USA) or a GGT inducer (sodium butyrate) for 3 h. After pretreatment, the cells were incubated with Nile Red-loaded tL-proxiRQ (75 µg/mL) for 0.5 or 1 h, and cellular uptake was subsequently analyzed using confocal microscopy. Red fluorescence intensity corresponding to Nile Red-loaded tL-proxiRQ was quantified using ImageJ software to evaluate differences in cellular uptake depending to GGT expression levels.

### Intracellular release of atRA and QM from tL-proxiRQ

To verify the intracellular release of atRA and QM from tL-proxiRQ, MCF-7 cells (1 × 10^6^ cells per 60 mm dish) were treated with tL-proxiRQ. After incubation for 12 h, the cells were collected and centrifuged to obtain cell pellets. The pellets were resuspended in 100 µL of deionized water and lysed by sonication. The resulting cell lysates were mixed with 900 µL of methanol and incubated at -20 °C for 1 h to induce protein precipitation, followed by centrifugation. The resulting supernatants were collected and subjected to LC-MS/MS analysis. Because QM is highly reactive and rapidly converted to its corresponding phenolic form under aqueous conditions, HBA was used as a stable surrogate marker to indirectly monitor intracellular QM generation. The collected supernatants were analyzed using an Acquity UPLC System (Waters, Milford, MA, USA) coupled to a Xevo^TM^ TQ-S triple quadrupole mass spectrometer (Waters, MS Technologies, Manchester, UK). For atRA analysis, chromatographic separation was performed using a ZORBAX SB-C8 Rapid Resolution Cartridge column (2.1 × 15 mm, 3.5 µm; Agilent Technologies, Santa Clara, CA, USA). The flow rate was maintained at 0.23 mL/min, the column temperature was set to 35 °C, and the injection volume was 10 µL. The mobile phase consisted of 0.1% formic acid in water as eluent A and 0.1% formic acid in acetonitrile as eluent B, with a total run time of 10 min. For HBA analysis, chromatographic separation was carried out using a Hydro-RP 80 Å column (Phenomenex, Torrance, CA, USA). The flow rate, column temperature, and injection volume were maintained at 0.23 mL/min, 35 °C, and 5 µL, respectively. The mobile phase consisted of 50 mM ammonium acetate in water as eluent A and 50 mM ammonium acetate in acetonitrile as eluent B, and the total run time was 10 min. Mass spectrometric detection was performed in the positive electrospray ionization mode (ESI +) with multiple reaction monitoring (MRM) for atRA analysis, while HBA analysis was conducted in the negative electrospray ionization mode (ESI -) with MRM. Tandem MS analysis was performed by monitoring the transition pairs of *m/z* 301.15 → 123.06 for atRA and *m/z* 122.93 → 104.77 for HBA. All data were acquired and processed using MassLynx 4.1 software.

### GSH-depleting ability of tL-proxiRQ

The GSH-depleting ability of tL-proxiRQ was evaluated using Ellman’s reagent. MCF-7 cells were seeded in 12-well plates at a density of 3.0 × 10^5^ cells per well and treated with various concentrations of tL-proxiRQ for 6 h. Following treatment, the cells were washed with PBS and lysed on ice using 100 µL of PRO-PREP Protein Extraction Solution C/T (iNtRON Biotechnology, Seongnam, Republic of Korea). The lysates were centrifuged at 13,000 × *g* for 30 min at 4 °C, and 10 µL of the supernatant was mixed with 50 µL of Ellman’s reagent. The remaining GSH levels were quantified by measuring the absorbance at 412 nm using a microplate reader (Synergy Mx, BioTek Instruments, Winooski, VT, USA).

### Determination of the intracellular ROS level

The intracellular ROS level was assessed by staining cells with DCFH-DA (2’,7’-dichlorofluorescein-diacetate; Sigma-Aldrich). MCF-7 cells were treated with various concentrations of tL-proxiRQ or with free atRA and preQM, individually or in combination, at molar concentrations equivalent to their respective contents in 100 µg/mL of tL-proxiRQ. After treatment, the culture medium was removed, and the cells were carefully washed with PBS. Cells were then incubated with DCFH-DA for 30 min at 37 °C, followed by washing with PBS. Fluorescence images were acquired using a confocal laser scanning microscope.

### Determination of mitochondrial damage and apoptotic cell death

To evaluate mitochondrial dysfunction and apoptotic cell death, MCF-7 cells were treated with various concentrations of tL-proxiRQ or with free atRA and preQM in combination at molar concentrations equivalent to 100 µg/mL of tL-proxiRQ. Following treatment, the cells were washed with PBS and stained using a JC-1 assay kit (Molecular Probes, Eugene, OR, USA) to access mitochondrial membrane potential (ΔΨ_m_). The stained cells were transferred to 5.0 mL round-bottom tubes and analyzed using a flow cytometer (CytoFLEX S, Beckman Coulter, Brea, CA, USA). For apoptotic cell death analysis, cells were stained with FITC-Annexin V and propidium iodide (PI) (BD Biosciences, San Diego, CA, USA) and incubated for 15 min at room temperature in the dark. The stained cells were then transferred to 5.0 mL round-bottom tubes and analyzed using a flow cytometer (FACSymphony A3, BD Biosciences, Franklin Lakes, NJ, USA). Both analyses were performed using a gating strategy for data collection and analysis. Specifically, primary gating was established based on forward scatter (FSC) and side scatter (SSC) profiles to separate single cell populations, thereby effectively excluding cell debris and doublets from the analysis.

### Cell viability assay

MCF-7, RAW 264.7, TCMK-1, HEK-293, and HUVEC cells were seeded in 24-well plates and treated with various concentrations of tL-proxiRQ. For comparison, MCF-7 cells were treated with free atRA and preQM at molar concentrations equivalent to 100 μg/mL of tL-proxiRQ. Following treatment, 100 µL of MTT solution (3-(4,5-dimethylthiazol-2-yl)-2,5-diphenyltetarazolium bromide) was added to each well and incubated for 3 h at 37 °C. After incubation, 1.0 mL of dimethyl sulfoxide (DMSO) was added to dissolve the resulting formazan crystals. The absorbance was measured at 570 nm using a microplate reader, and cell viability was calculated by comparing the absorbance values to those of PBS-treated control cells.

To investigate the involvement of intracellular ROS in tL-proxiRQ-induced cytotoxicity, an additional MTT assay was performed with *N*-acetyl-L-cysteine (NAC) pretreatment in MCF-7 cells. MCF-7 cells were pretreated with NAC at a concentration of 1 mM for 2 h. The cells were subsequently treated with tL-proxiRQ at various concentrations and incubated for an additional 24 h. Cell viability was then evaluated using the same MTT assay procedure described above.

### Cell cycle analysis

MCF-7 cells were seeded into 60-mm dishes at a density of 1 × 10^6^ cells per dish and treated for 16 h with tL-proxiRQ at concentrations of 25, 50 or 100 μg/mL, or with free atRA and preQM in combination at the molar concentrations equivalent to100 μg/mL of tL-proxiRQ. Following treatment, the cells were washed with PBS and harvested by centrifugation. The collected cells were fixed in 70% ethanol at -20 °C for 30 min. After fixation, the cells were washed again with PBS and centrifuged. The cell pellets were incubated in staining buffer containing RNase A (100 μg/mL) and PI (20 μg/mL) at 37 °C for 30 min. Cell cycle distribution was analyzed using a flow cytometer. A consistent gating strategy was applied for accurate quantification. To remove cell debris and doublets, single cells were isolated based on FSC and SSC profiles so that DNA content analysis was performed only on the singlet population.

### Quantitative polymerase chain reaction (qPCR)

MCF-7 cells were seeded in 12-well plates at a density of 3 × 10^5^ cells per well and incubated overnight. Cells were then treated with various concentrations of tL-proxiRQ for indicated time points. Following treatment, the culture medium was removed, and the cells were washed twice with PBS. The 500 μL of TRIzol Reagent (Invitrogen) was added to each well. Subsequently, chloroform was added, and the mixture was gently vortexed for 10 s, repeated three times, and incubated at room temperature for 5 min. Samples were centrifuged at 13,000 × *g* for 20 min at 4 °C, and the aqueous phase was collected for RNA extraction. Total RNA was quantified using a BioSpectrometer Kinetic (Eppendorf, Hamburg, Germany), and samples with an A_260_/A_280_ ratio between 1.8 and 2.1 were used for downstream analysis. First-strand complementary DNA (cDNA) was synthesized using the Thermo cDNA Synthesis Kit (Thermo Fisher Scientific) on a Takara PCR Thermal Cycler (Takara Bio, Shiga, Japan). Quantitative PCR was performed using a StepOnePlus^TM^ Real-Time PCR system (Applied Biosystems, Foster City, CA, USA). Each 15 μL reaction contained 2.0 μL of cDNA, 0.5 μL each of forward and reverse primers (0.2 μM final concentration), 7.5 μL of PowerUp^TM^ SYBR^TM^ Green Master Mix (Applied Biosystems), and 4.5 μL of nuclease-free water.

The qPCR cycling conditions were as follows: holding stage at 50 °C for 2 min and initial denaturation at 95 °C for 10 min, followed by 40 cycles of denaturation at 95 °C for 15 s and annealing/extension at 60 °C for 1 min. Melting curve analysis was performed by increasing the temperature from 60 °C to 95 °C with continuous fluorescence acquisition, after a denaturation step at 95 °C for 15 s and a pre-hold at 60 °C for 1 min. Primer sequences are listed in Supplementary Table S3 and were purchased from Bioneer (Daejeon, Republic of Korea). All reactions were optimized to ensure exponential amplification, and *TBP* was used as internal reference genes.

### Western blot analysis

MCF-7 cells were seeded in 6-well plates at a density of 5 × 10^5^ cells per well and incubated overnight. The cells were then treated with either tL-proxiRQ at various concentrations or a combination of free atRA and preQM at molar concentrations equivalent to 100 µg/mL of tL-proxiRQ, for the indicated time points. After treatment, the cells were washed three times with PBS and harvested by centrifugation at 3,500 × *g* for 5 min at 4 °C. The supernatant was discarded, and 100 µL of PRO-PREP Protein Extraction Solution C/T was added to each pellet. Samples were incubated on ice for 15 min and then sonicated to lyse the cells. The lysates were centrifuged at 13,000 × *g* for 30 min at 4 °C, and the supernatants were collected for further analysis.

Protein concentrations were determined using the Bradford assay. Equal amounts of protein were loaded onto 13% and 8% polyacrylamide gels and subjected to SDS-PAGE. Proteins were then transferred to polyvinylidene difluoride (PVDF) membranes. The membranes were blocked with 5% skim milk in TBST (Tris-buffered saline containing 0.1% Tween-20; KBIO Company, Seoul, Republic of Korea) and incubated overnight at 4 °C with primary antibodies diluted in TBST containing 0.5% BSA, with gentle rocking. After three washes with TBST (15 min each), the membranes were incubated with HRP-conjugated secondary anti-IgG antibodies at room temperature for 1 h with rocking. Immunoreactive protein bands were visualized using the EZ-Western Lumi Pico Alpha detection system (DoGenBio, Seoul, Republic of Korea), and images were acquired using a FUSION FX system (Vilber Lourmat, Collégien, France). After target protein detection, membranes were stripped using Easter-Blot Western Blot Stripping Buffer (BIOMAX, Seoul, Republic of Korea), and subsequently re-probed for loading control proteins using the same blocking and immunoblotting procedure. Densitometric quantification of the protein bands was performed using ImageJ software. The target protein expression levels were calculated by normalizing the intensity of each target band to the intensity of the corresponding loading control, and the results are presented as relative values compared to the control group. The list of antibodies used is provided in Supplementary Table S3.

### Blood circulation analysis of L-proxiRQ and tL-proxiRQ

To evaluate the blood circulation profiles of L-proxiRQ and tL-proxiRQ, IR780-loaded liposomes were intravenously administered to rats *via* tail vein injection. At predetermined time points (0.5, 1, 3, 6, 9, 12, 24, 48, 72, and 96 h), blood samples were collected by retro-orbital bleeding. The collected blood samples were transferred into tubes containing sodium citrate as an anticoagulant and stored at -80 °C until analysis. The concentration of circulating liposomes was quantified by measuring the fluorescence intensity of IR780, and blood concentration-time curves were generated. The circulation half-life was estimated from the resulting fluorescence intensity-time profiles, and the area under the curve (AUC_0.5–96 h_) was calculated using the linear trapezoidal method to assess the overall blood exposure of each formulation. Blood concentrations were expressed as the percentage of the injected dose per milliliter (%ID/mL).

### Xenograft mouse model

To establish a xenograft mouse model, MCF-7 cells (4.0 × 10^6^ cells in 100 μL of PBS) were subcutaneously injected into the left flank of 8-week-old nude BALB/c mice (Orient Bio, Republic of Korea). To evaluate the tumor-targeting capability of tL-proxiRQ, Nile Red-loaded L-proxiRQ or tL-proxiRQ was administered *via* tail vein injection. Fluorescence images were acquired using a fluorescence imaging system (Fluor i, NeoScience, Republic of Korea) under red-light excitation. Once tumors reached approximately 80 mm^3^ in volume, the mice were randomly assigned to six groups. To assess therapeutic efficacy, mice were intravenously injected every three days with free atRA (3.7 mg/kg), preQM (2.9 mg/kg), or a combination of atRA and preQM, at a dose equivalent to the amounts contained in 20 mg/kg of tL-proxiRQ, or with tL-proxiRQ (10 or 20 mg/kg). Tumor volumes were measured every three days for up to 25 days using a caliper and calculated according to the formula: volume = L × W^2^ × 0.52, where L and W represent tumor length and width, respectively. Body weights were also recorded every three days throughout the study period. On day 25, the mice were sacrificed for histological analysis. Tumors were excised, and major organs (heart, lungs, liver, kidneys, and spleen) were collected. All tissues were fixed in 10% neutral buffered formalin and embedded in paraffin. All animal experiments were conducted in accordance with the guidelines approved by the Institutional Animal Care and Use Committee of Jeonbuk National University (JBNU 2022-069), Republic of Korea.

### Biosafety of tL-proxiRQ

To evaluate the biosafety of tL-proxiRQ, healthy mice were intravenously administered tL-proxiRQ at a dose of 50 mg/kg every other day for 10 days. On day 12, blood and major organs (heart, lungs, liver, kidneys, and spleen) were collected. Serum levels of alanine transaminase (ALT) and aspartate transaminase (AST), blood urea nitrogen (BUN), and creatinine (Cr) were quantified using ALT/AST assay kit (Asan Pharmaceutical, Seoul, Republic of Korea), a BUN assay kit (Abbexa, Cambridge, UK), and a creatinine assay kit (Abcam, Cambridge, UK), respectively. Harvested organs were fixed in 10% neutral buffered formalin and embedded in paraffin. Tissue sections (5 μm) were prepared, stained with hematoxylin and eosin (H&E), and examined under an inverted microscope (Taeshin Bio, Seoul, Republic of Korea).

### Statistical analysis

Statistical analyses were performed using GraphPad Prism 8 (GraphPad Software, San Diego, CA, USA). Statistical comparisons between two independent groups were performed using an unpaired two-tailed Student’s *t*-test. For multiple comparisons among three or more groups, data were analyzed using one-way analysis of variance (ANOVA) followed by Tukey’s multiple comparisons test to determine statistical significance between groups. *P*-values less than 0.05 were considered statistically significant. All data are presented as the mean ± standard deviation (s.d.).

## Results

### Synergistic anticancer effect of atRA and QM

**Figure 1:**
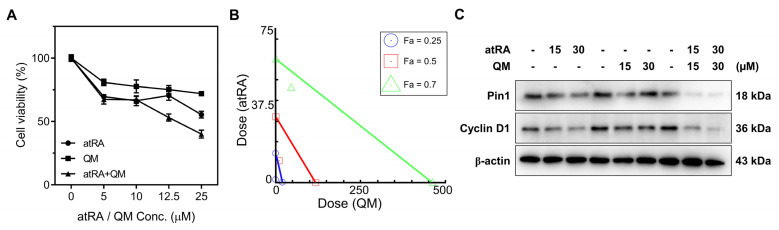
Synergistic cytotoxicity and Pin1 suppression by the combined treatment with atRA and preQM. (A) Cell viability assay of MCF-7 cells treated with atRA, preQM, or their combination. Values are mean ± s.d. (n = 3). (B) Isobologram-based combination index analysis of atRA and preQM. (C) Western blot analysis of Pin1 and Cyclin D1 (loading control: β-actin).

We hypothesized that GSH-depleting QM would potentiate the anticancer activity of atRA by driving excessive accumulation of ROS, thereby reinforcing atRA-mediated Pin1 suppression and promoting cancer cell death 5, 20, 32. To test this hypothesis, MCF-7 cells were co-treated with atRA and a QM precursor (preQM), which is enzymatically converted by esterase to generate two QM intermediates 33 and the cytotoxic effects were compared with those of individual agents. Cell viability assays revealed that the co-treatment with atRA and preQM exhibited higher cytotoxicity than either agent alone (Figure 1A). Isobologram analysis using CompuSyn software revealed that the combination index (CI) values were below 1 at fractional affected (Fa) values of 0.25, 0.5, and 0.7, confirming a synergistic interaction between atRA and QM (Figure 1B). Western blot analysis showed that the combination therapy more effectively suppressed the expression of Pin1 and its downstream target, Cyclin D1, compared to single treatments (Figure 1C and S1). These results support the notion that atRA-induced ROS increase is further amplified by QM-mediated GSH depletion, leading to ROS accumulation, which in turn enhances Pin1 inhibition, forming a synergistic anticancer mechanism. The observed synergy provides a compelling rationale for developing a mutual prodrug strategy that enables synchronized intracellular release.

### Synthesis and characterization of proxiRQ

**Figure 2:**
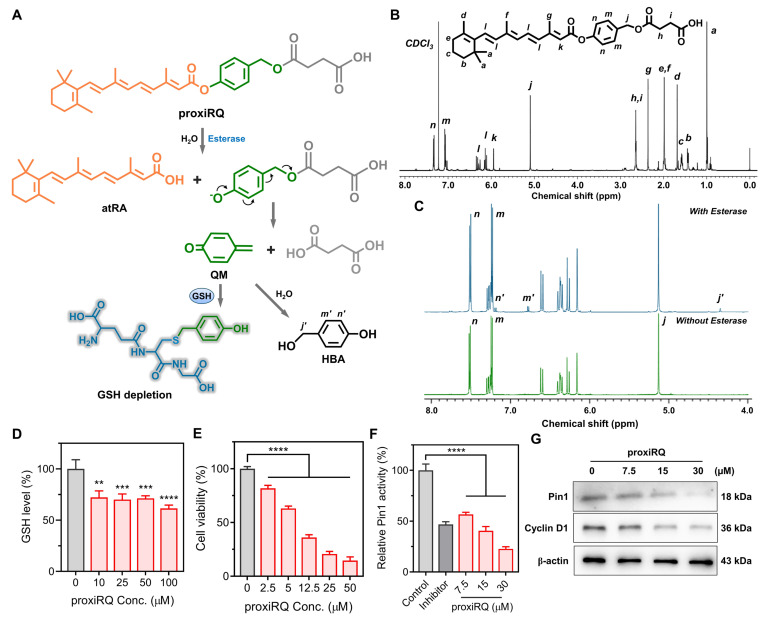
proxiRQ as a dual-functional mutual prodrug of atRA and QM rationally designed for oxidative stress amplification and Pin1 inhibition. (A) Esterase-mediated degradation of proxiRQ. (B) ^1^H NMR spectrum of proxiRQ recorded in CDCl_3_ (500 MHz). (C) ^1^H NMR spectra of proxiRQ with or without esterase treatment. Prior to analysis, samples were lyophilized and re-dissolved in DMSO-d_6_. (D) GSH depleting ability of proxiRQ. ^**^P < 0.01, ^***^P < 0.001, ^****^P < 0.0001 relative to untreated group (0 μM proxiRQ). Values are mean ± s.d. (n = 3). (E) Cytotoxicity of proxiRQ against MCF-7 cells. ^****^P < 0.0001. Values are mean ± s.d. (n = 3). (F) Inhibition of Pin1 catalytic activity by proxiRQ. ^****^P < 0.0001. Values are mean ± s.d. (n = 3). (G) Western blot analysis of Pin1 and Cyclin D1 (loading control: β-actin).

Encouraged by the synergism between atRA and QM, we developed a mutual prodrug, proxiRQ, designed to deliver atRA and QM concurrently. proxiRQ was synthesized by replacing the carboxylic acid group of atRA with an aryl ester and appending a terminal carboxyl group to impart amphiphilicity (Figure 2A and S2). This architecture enables proxiRQ to act as both a therapeutic and a structural component in liposomal formulations. The chemical structure of proxiRQ was confirmed by ^1^H NMR (Figure 2B). The aromatic protons of the phenyl ring (*n*, *m*) appeared at δ = 7.4 and 7.1. The β-vinyl proton (=CH-, *k*) of the α,β-unsaturated ester moiety was observed at δ = 5.9. The benzylic methylene protons (-Ph-CH_2_-O-, *j*) were identified at δ = 5.1. The methylene protons of the butanoic acid chain adjacent to the terminal carboxylic acid (*h*, *i*) were detected at δ = 2.7. Mass spectrometric analysis (LC-MS/MS ESI - mode) revealed a dominant [M - H] - ion at *m/z* 504.5, confirming the molecular weight of proxiRQ (Figure S3A). Upon esterase treatment, proxiRQ underwent hydrolysis to release both atRA and QM (Figure 2C and S3B). Since QM rapidly reacts with water to form HBA, its release was indirectly validated using two complementary methods. First, ^1^H NMR analysis identified the aromatic protons of HBA (*n´, m´*) at δ = 7.2 and 6.7, respectively. Second, this conversion was further corroborated by detecting the specific HBA fragment at *m/z* 168.3 ([M + HCOO] -) in the presence of formic acid. Concurrently, the corresponding atRA fragment ion appeared at *m/z* 254.3 ([M - H- CO_2_] -), confirming the simultaneous liberation of both active components.

To determine whether these structural features of proxiRQ lead to functional biological activity, we evaluated its three key aspects: GSH-depleting ability, ROS-mediated apoptosis, and Pin1 inhibition. As expected, esterase-activated proxiRQ effectively depleted GSH in a concentration-dependent manner, reflecting the generation of electrophilic QM (Figure 2D). Moreover, proxiRQ exhibited markedly increased cytotoxic effects against MCF-7 cells compared to co-treatment with atRA and preQM (Figure 1A and 2E), suggesting that the concurrent delivery of atRA and QM from the prodrug contributes to enhanced anticancer efficacy. Furthermore, enzymatic analysis revealed that Pin1 activity decreased to approximately 20% of the untreated control in a concentration-dependent manner upon proxiRQ treatment (Figure 2F), which implies that the essential catalytic function of Pin1 was inhibited similarly to or more potently than that of the control inhibitor. In parallel, Western blot analysis showed that proxiRQ also markedly suppressed Pin1 expression, thereby reducing Cyclin D1 expression (Figure 2G and S4). Collectively, these results indicate that proxiRQ successfully recapitulates the synergistic effects of atRA and QM.

### Preparation and characterization of tL-proxiRQ

proxiRQ possesses an amphiphilic structure with a hydrophobic unsaturated alkyl tail and a polar carboxyl headgroup. This molecular design was expected to promote co-assembly with DPPC, a representative phospholipid containing two saturated alkyl chains and a zwitterionic headgroup, commonly used for forming stable bilayers. L-proxiRQ was prepared by reverse-phase evaporation method using proxiRQ and DPPC in a molar ratio of 4:6. The resulting liposomes exhibited a spherical morphology with a hydrodynamic diameter of approximately 130 nm, as confirmed by TEM and dynamic light scattering (DLS) analyses (Figure S5). These findings suggest that the amphiphilic structure of proxiRQ facilitates cooperative packing with DPPC, promoting stable bilayer formation. In contrast, liposomes could not be obtained using atRA under the identical conditions (Figure S6A), highlighting the necessity of the optimized amphiphilicity of proxiRQ. Similarly, co-formulation with the cationic lipid DOTAP failed to form bilayer structures (Figure S6B) due to electrostatic disruption and unfavorable packing geometry.

**Figure 3:**
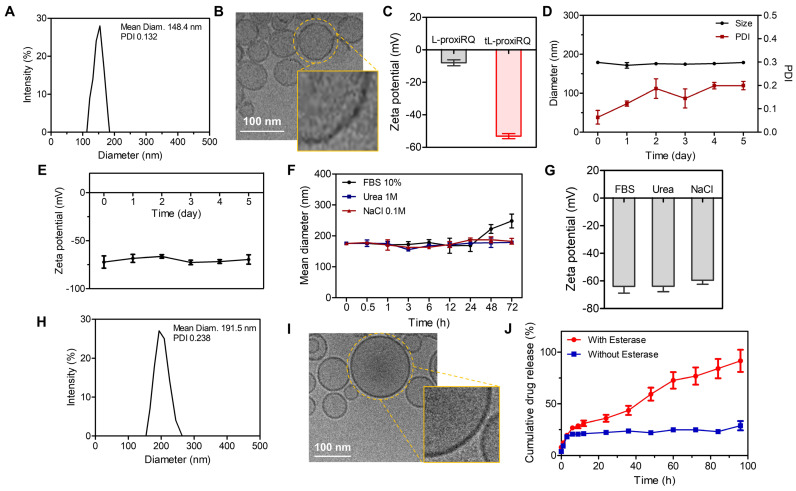
Characterization of tL-proxiRQ. (A) Size distribution of tL-proxiRQ. (B) Cryo-TEM image showing the multilamellar vesicle morphology of tL-proxiRQ. (C) Zeta potential of L-proxiRQ and tL-proxiRQ dispersed in diluent PBS. Values are mean ± s.d. (n = 4). (D) Change in hydrodynamic size and PDI of tL-proxiRQ. Values are mean ± s.d. (n = 4). (E) Change in zeta potential of tL-proxiRQ. Values are mean ± s.d. (n = 4). (F) Colloidal stability of tL-proxiRQ under conditions with physiologically relevant stressors. Values are mean ± s.d. (n = 3). (G) Zeta potential of tL-proxiRQ measured at 72 h under conditions with physiologically relevant stressors. Values are mean ± s.d. (n = 3). (H) Size distribution and (I) cryo-TEM image of tL-proxiRQ after one month of storage at 4 °C. (J) Cumulative QM release from tL-proxiRQ in the presence or absence of esterase. Values are mean ± s.d. (n = 3).

tL-proxiRQ was obtained by additionally introducing γPGA into L-proxiRQ for tumor targeting. γPGA is a biodegradable polyanion that mimics γ-glutamyl substrates and can be selectively recognized by GGT, which is overexpressed in various tumors. γPGA was adsorbed onto L-proxiRQ through electrostatic interactions with the quaternary ammonium groups of DPPC. This modification did not significantly alter the hydrodynamic diameter, as determined by DLS (Figure 3A). Furthermore, cryo-TEM imaging revealed that tL-proxiRQ retained a spherical, multilamellar vesicle structure with a bilayer thickness of approximately 5 nm (Figure 3B). Successful surface modification was verified by zeta potential measurements, which showed a significant reduction compared with L-proxiRQ (Figure 3C). To evaluate formulation efficiency, the DLC and estimated EE of tL-proxiRQ were determined by ^1^H NMR analysis (Figure S7). Based on the integration ratio of the characteristic benzyl protons signal of proxiRQ to the the *N*-methyl protons (–C*H*_2_-N^+^-) signal of DPPC, the molar fraction of proxiRQ in the liposomal formulation was determined to be approximately 37%. Additionally, using the precise stoichiometry obtained from the NMR data, the estimated EE was calculated to be approximately 88.2%. These quantitative results demonstrate that the amphiphilic mutual prodrug strategy enables a highly efficient manufacturing process that ensures exceptionally high drug content and minimal drug waste. In addition, tL-proxiRQ displayed excellent colloidal stability. The liposomes maintained consistent size, polydispersity index (PDI) and zeta potential for 5 days in diluent PBS, and no significant changes in zeta potential values were observed (Figure 3D-E). Under physiologically relevant stressors including 10% FBS, 1 M urea, or 0.1 M NaCl, tL-proxiRQ maintained stable particle sizes for 72 h (Figure 3F), and the zeta potential remained negative after 72 h, except for shifting slightly further into the negative range (Figure 3G). Even after one month of storage at 4 °C, the liposomes retained a spherical morphology and the particle size increased slightly (Figure 3H-I). Notably, this long-term stability was achieved without cholesterol, which is typically required to rigidify conventional liposomal membranes. This finding underscores the inherent structural complementarity and self-assembling capability of DPPC and proxiRQ.

Mechanistically, proxiRQ was designed to release atRA and a QM intermediate *via* esterase-catalyzed hydrolysis. QM can deplete intracellular GSH or be hydrolyzed to HBA. The esterase-responsive drug release behavior of tL-proxiRQ was indirectly assessed by quantifying the cumulative HBA production as a surrogate marker (Figure 3J). In the absence of esterase, drug release was minimal, reaching approximately 20% after 6 h and remaining largely unchanged through 96 h. In contrast, exposure to esterase induced a time-dependent release, reaching approximately 50% after 48 h and greater than 80% after 96 h. These results demonstrate that the prodrug design enables efficient esterase-responsive release, and that tL-proxiRQ serves as a smart delivery system that can be activated on-demand in the esterase-rich tumor.

### GGT-mediated cellular uptake and endosomal escape of tL-proxiRQ

**Figure 4:**
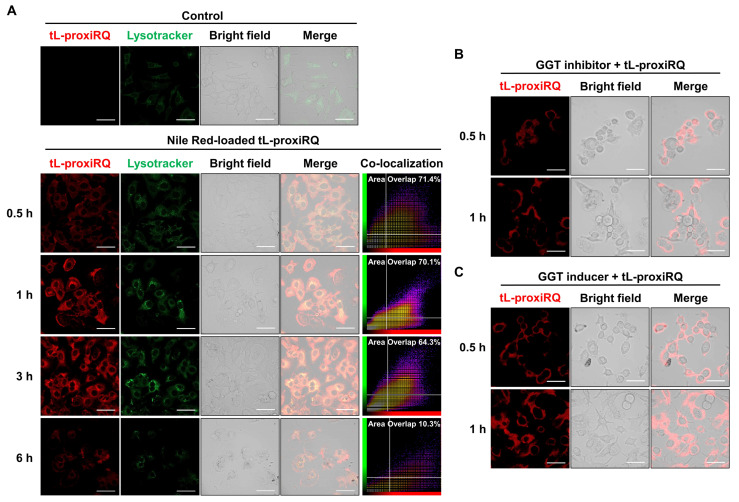
GGT-mediated cellular uptake and endosomal escape of tL-proxiRQ. (A) Time-dependent cellular uptake and endosomal escape of Nile Red-loaded tL-proxiRQ in MCF-7 cells. Scale bar is 50 µm. Cellular uptake of tL-proxiRQ after treatment with (B) GGT inhibitor or (C) GGT inducer. Scale bar is 50 µm.

The intracellular trafficking and endosomal escape of tL-proxiRQ were investigated in MCF-7 cells using confocal laser scanning microscopy. Cells were incubated with Nile Red-loaded tL-proxiRQ for 0.5, 1, 3, and 6 h and the red fluorescence signal was monitored along with the green signal from LysoTracker (Figure 4A). At early time points (0.5 and 1 h), robust colocalization of the red and green fluorescence was observed, indicating that tL-proxiRQ accumulated primarily within endo/lysosomal compartments. At 3 h, partial separation of the red signal from the lysosomal marker was detected, suggesting that endosomal escape had initiated. At 6 h, the red fluorescence became more diffuse and its intensity weakened, which is likely due to the dispersion of the released payload into the aqueous cytosol and the polarity-sensitive fluorescence quenching of Nile Red in hydrophilic environments 34, 35. Concurrently, a marked reduction in the green LysoTracker fluorescence was also observed after 6 h. Because LysoTracker is highly dependent on the acidic pH of the intact endosomes/lysosomes, this simultaneous quenching indicates that tL-proxiRQ successfully disrupted the endosomal membranes. To quantitatively validate the visual observations, co-localization indices were analyzed (Table S1). The area overlap (%) remained high (> 64%) until 3 h, but decreased sharply to 10.3% after 6 h, confirming the physical spatial separation and cytosolic translocation of tL-proxiRQ. Furthermore, Spearman’s rank coefficient dropped from 0.885 at 3 h to 0.592 at 6 h, verifying the definitive disruption of signal colocalization. Although Pearson’s coefficient remained high even after 6 h, this is because the ratio of the two signals still maintains a linear correlation within the minor 10.3% region. Collectively, the temporal shift from initial signal colocalization to the subsequent quenching of both signals is further supported by the quantitative co-localization analysis, which confirms the efficient endosomal escape and cytosolic delivery of tL-proxiRQ.

Given that tL-proxiRQ was designed as a GGT-targeting liposomal formulation, the effect of γPGA-mediated GGT recognition on cellular uptake was further evaluated. MCF-7 cells were pretreated with GGsTop (GGT inhibitor) or sodium butyrate (GGT inducer) for 3 h 36, 37, followed by incubation with Nile Red-loaded tL-proxiRQ for 0.5 or 1 h (Figure 4B-C). In GGT-inhibited cells, intracellular red fluorescence intensity was significantly reduced at both time points, indicating decreased cellular uptake. Conversely, GGT-induced cells exhibited markedly higher red fluorescence than GGT-inhibited cells, suggesting that increased GGT expression facilitates tL-proxiRQ uptake into cells (Figure S9). Collectively, these results demonstrate that tL-proxiRQ is efficiently internalized into tumor cells through GGT-targeted mechanisms and successfully escapes from the endosomal/lysosomal compartments into the cytosol. The γPGA surface decoration enables receptor-mediated uptake *via* GGT, contributing to both selectivity and uptake efficiency.

### Intracellular release of atRA and QM from tL-proxiRQ

**Figure 5:**
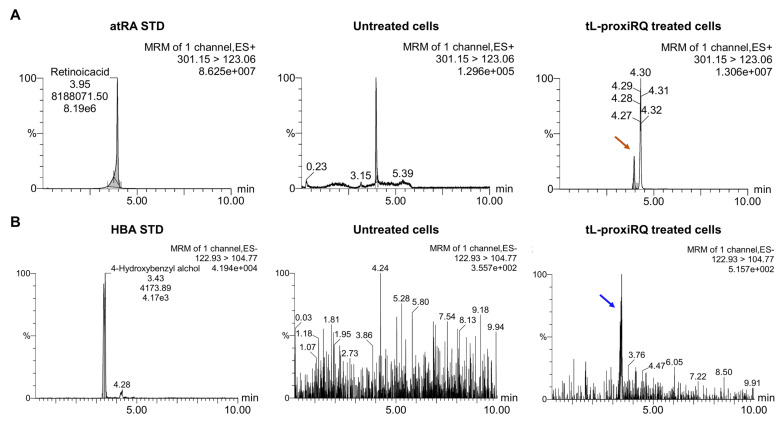
LC-MS/MS analysis of intracellular (A) atRA and (B) QM released from tL-proxiRQ. Arrows indicate the detection of standard atRA and HBA, used as a surrogate for QM.

To examine the intracellular degradation of tL-proxiRQ and the subsequent release of atRA and QM, MCF-7 cells treated with tL-proxiRQ for 12 h were collected, lysed, and subjected to LC-MS/MS analysis (Figure 5). Given the highly reactive and transient nature of QM, which is rapidly converted to its phenolic form under aqueous conditions, HBA was employed as a surrogate marker to indirectly reflect intracellular QM generation. In cells exposed to tL-proxiRQ, distinct and clear peaks corresponding to atRA and HBA were detected, confirming the intracellular presence of both components after treatment. In contrast, no peaks corresponding to atRA or HBA were detected in untreated control cells, indicating that the detected signals originated from tL-proxiRQ-derived species rather than natural intracellular components. The simultaneous detection of atRA and HBA in cell lysates suggests that tL-proxiRQ is effectively internalized into MCF-7 cells and degraded in the intracellular environment.

### Prooxidant activity and redox disruption induced by tL-proxiRQ

**Figure 6:**
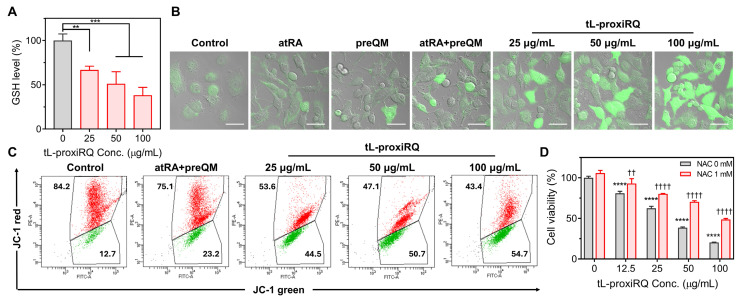
Oxidative stress-mediated cellular perturbations induced by tL-proxiRQ. (A) Intracellular GSH levels measured after the treatment with various concentrations of tL-proxiRQ. ^**^P < 0.01, ^***^P < 0.001. Values are mean ± s.d. (n = 3). (B) DCFDA-based detection of intracellular ROS levels following treatment with atRA, preQM, their combination, or tL-proxiRQ. Scale bar is 50 µm. (C) Flow cytometric analysis of mitochondrial membrane potential after treatment with tL-proxiRQ. Cells were stained with JC-1 dye. (D) Cell viability assessment of tL-proxiRQ-treated cells with or without NAC pretreatment.^ ****^P < 0.0001 relative to the untreated group (0 μg/mL tL-proxiRQ) in the absence of NAC; ^††^*P* < 0.01, ^††††^*P* < 0.0001 relative to the untreated group (0 μg/mL tL-proxiRQ) in the presence of NAC. Values are mean ± s.d. (n = 3).

To investigate the redox-based cytotoxic mechanism of tL-proxiRQ, a series of redox-related assays were performed. First, the intracellular GSH depleting activity was assessed to evaluate its capacity to disrupt the antioxidant defense system (Figure 6A). tL-proxiRQ treatment led to a concentration-dependent depletion of intracellular GSH, indicating its potential to impair antioxidant defense system. Next, intracellular ROS levels were measured using DCFDA to examine the prooxidant effect. While free atRA, preQM, or their combination, at a molar concentration equivalent to 100 µg/mL of tL-proxiRQ, elicited negligible ROS generation, tL-proxiRQ treatment markedly elevated intracellular ROS levels (Figure 6B and S10A). These results suggest that the mutual prodrug structure enhances intracellular redox disruption compared to the unlinked components. Since excessive ROS accumulation can impair mitochondrial integrity, changes in ΔΨ_m_ were evaluated using JC-1 staining and flow cytometry (Figure 6C and S10B). A pronounced increase in green fluorescence corresponding to JC-1 monomers was observed after tL-proxiRQ treatment, indicating a loss of ΔΨ_m_ and mitochondrial dysfunction. To validate whether tL-proxiRQ-mediated ROS accumulation contributes to cytotoxicity, we performed a rescue experiment using the antioxidant NAC. NAC pretreatment partially reversed the cytotoxic effect of tL-proxiRQ, supporting the involvement of ROS-mediated mechanisms in the apoptosis-inducing activity of tL-proxiRQ (Figure 6D). Collectively, these findings demonstrate that tL-proxiRQ depletes GSH and induces ROS accumulation, disrupting intracellular redox balance and leading to mitochondrial dysfunction and oxidative stress-mediated cell death.

### Anticancer activities of tL-proxiRQ

**Figure 7:**
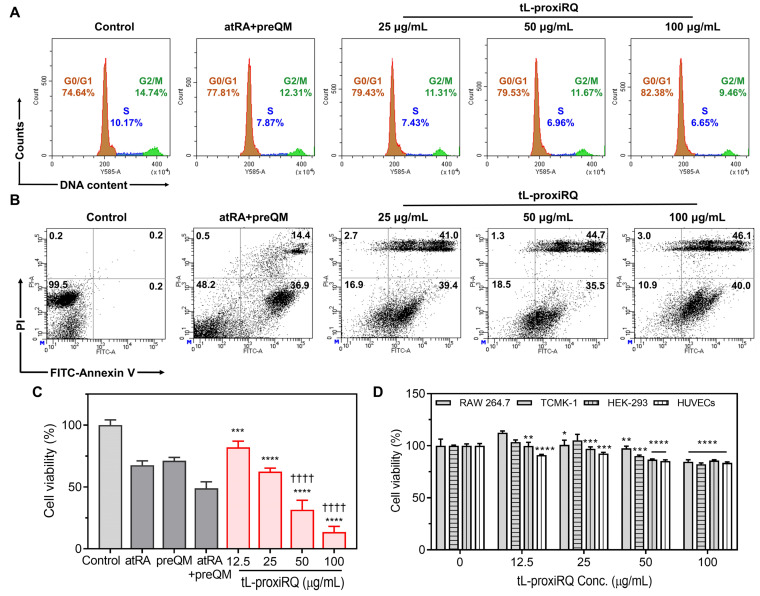
Anticancer activity of tL-proxiRQ. (A) Cell cycle arrest assay after the treatment with the combination of atRA and preQM, and various concentrations of tL-proxiRQ. (B) Flow cytometric analysis of cells stained with FITC-Annexin V and PI. (C) Cytotoxicity in MCF-7 cells treated with atRA, preQM, their combination, and various concentrations of tL-proxiRQ. ^***^P < 0.001, ^****^P < 0.0001 relative to control. ^††††^*P* < 0.0001 relative to atRA + preQM. Values are mean ± s.d. (n = 4). (D) Cytotoxicity of tL-proxiRQ in normal cells. ^*^P < 0.05, ^**^P < 0.01, ^***^P < 0.001, ^****^P < 0.0001 relative to the untreated group (0 μg/mL tL-proxiRQ) for each respective cell line. Values are mean ± s.d. (n = 4).

ROS accumulation and GSH depletion are known to inhibit the activity of Pin1, a key regulator of cell cycle progression and tumor growth 13, 38. Inhibition of Pin1 disrupts the function of multiple cell-cycle regulatory proteins, leading to G1 phase arrest, decreased S phase population, and the suppression of cancer cell proliferation 39, 40. To investigate the effect of tL-proxiRQ on cell cycle dynamics, flow cytometric analysis of DNA content was performed following PI staining (Figure 7A and S11A). Treatment with tL-proxiRQ markedly increased the proportion of cells in the G1 phase while decreasing the S phase population, indicative of impaired DNA synthesis and G1 phase cell cycle arrest. Next, apoptosis induction was evaluated by dual staining with FITC-conjugated Annexin V and PI. A gating strategy was applied to exclude debris and doublets, ensuring reliable quadrant-based discrimination of apoptotic stages. tL-proxiRQ treatment resulted in a significantly higher proportion of cells in the upper right quadrant (Annexin V^+^/PI^+^) compared to both control and co-treatment of atRA and preQM (Figure 7B and S11B), indicating superior apoptosis-inducing capability. The cytotoxicity of tL-proxiRQ was further evaluated using the MTT assay in MCF-7 cells. tL-proxiRQ exhibited more potent cytotoxic effects than atRA, preQM, or their co-treatment, at a same molar concentration as 100 µg/mL of tL-proxiRQ (Figure 7C). Furthermore, to evaluate the broad applicability of tL-proxiRQ, we investigated its cytotoxic potential in various cancer cell lines, including Huh7 (human liver cancer), A549 (human lung cancer), and CT26 (mouse colorectal cancer). As shown in Figure S12, tL-proxiRQ induced dose-dependent significant cytotoxicity in all test cell lines. In contrast, tL-proxiRQ exhibited no significant cytotoxicity at any concentration in normal cell lines, including RAW 264.7, TCMK-1, HEK-293, and HUVEC cells (Figure 7D). This demonstrates that tL-proxiRQ possesses selective toxicity toward cancer cells. To determine whether this lack of cytotoxicity in macrophages was simply due to low intracellular uptake, the cellular uptake of IR780-loaded tL-proxiRQ was evaluated using flow cytometry (Figure S13). MCF-7 cells exhibited a rapid and extensive uptake pattern, whereas RAW 264.7 macrophages showed a somewhat slower but distinct uptake pattern. Taken together, these results indicate that tL-proxiRQ efficiently penetrates cancer cells, disrupting redox homeostasis, inducing G1 phase cell cycle arrest, and promoting late-stage apoptosis without affecting normal cells. These selective and multifaceted mechanisms suggest that tL-proxiRQ possesses therapeutic potential as a self-synergizing mutual prodrug for targeted cancer therapy.

### Pin1 inactivation and apoptosis induced by tL-proxiRQ

**Figure 8:**
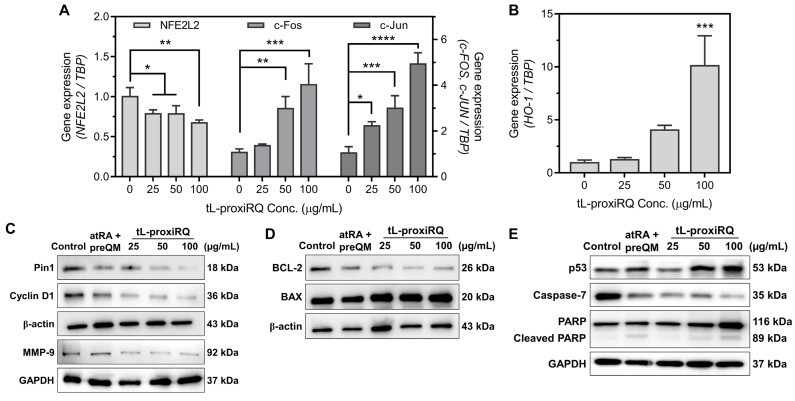
Pin1 inactivation and apoptotic signaling induced by dual redox modulation of tL-proxiRQ. qPCR analysis of (A) *NFE2L2*, *c-Fos*, *c-Jun*, and (B) *HO-1* mRNA expression (reference gene: *TBP*). ^*^P < 0.05, ^**^P < 0.01, ^***^P < 0.001, ^****^P < 0.0001 relative to untreated group (0 μg/mL tL-proxiRQ). Values are mean ± s.d. (n = 3). Western blot analysis of (C) Pin1, Cyclin D1 (loading control: β-actin), MMP-9 (loading control: GAPDH), (D) BCL-2, and BAX protein expression (loading control: β-actin). (E) Western blot analysis of p53, Caspase-7, PARP, and cleaved PARP protein expression (loading control: GAPDH).

To elucidate the cellular effects of tL-proxiRQ-mediated dual redox modulation, we analyzed changes in the expression of genes and proteins related to oxidative stress and apoptosis using qPCR and Western blot analysis. Gene expression profiling revealed a moderate downregulation in mRNA expression of *NFE2L2*, a master regulator of antioxidant defense, while the levels of *c-Fos* and *c-Jun*, key components of the AP-1 transcription factor complex, were significantly upregulated (approximately 4- to 5-fold) (Figure 8A). These results suggest that prolonged oxidative stress induced by tL-proxiRQ compromises the *NFE2L2*-mediated antioxidant defense system and simultaneously activates the AP-1 signaling pathway 41-43. In addition, abnormally increased expression of heme oxygenase-1 (*HO-1*) was observed (Figure 8B), which may reflect a terminal cellular response aimed at cellular adaptation *via* AP-1-mediated transcriptional activation 44, 45 and/or engagement of alternative survival pathways such as antioxidant defense and drug resistance 46, 47.

To support the hypothesis that excessive ROS production and Pin1 inhibition contribute to apoptotic cell death, we analyzed changes in the protein levels of key regulators of cell fate. Notably, Pin1 expression was decreased, likely due to direct binding of atRA and/or ROS-mediated oxidation at its catalytically active Cys113 residue (Figure 8C and S14A). Correspondingly, Cyclin D1, a substrate of Pin1, was also downregulated, indicating that cell cycle progression was inhibited (Figure S14B) 38-40. In addition, the expression of MMP-9, another important Pin1 substrate that promotes tumor metastasis by inducing extracellular matrix degradation, was significantly reduced (Figure S16C). Concurrently, p53 was stabilized (Figure 8E and S16A), which reflects the activation of the tumor suppressor induced by stress. Analysis of the expression profile of apoptosis-related proteins revealed a decrease in the anti-apoptotic BCL-2 and an increase in the pro-apoptotic BAX, suggesting activation of the mitochondrial apoptotic pathway (Figure 8D and S15). Furthermore, the decreased levels of full-length Caspase-7, an apoptotic executioner caspase, were consistent with its cleavage and activation. The presence of cleaved PARP, indicating progression to the final stage of apoptosis, was further confirmed (Figure S16B and S16E). Collectively, these findings suggest that dual ROS generation by tL-proxiRQ disrupts redox homeostasis and the antioxidant defense mechanism, leading to inactivation of Pin1 and consequent activation of mitochondria-mediated apoptotic signaling. These results provide a mechanistic basis for designing proxiRQ as a self-synergistic mutual prodrug that combines atRA and QM to exert multifaceted anticancer effects.

### Biodistribution and blood pharmacokinetics of tL-proxiRQ

**Figure 9:**
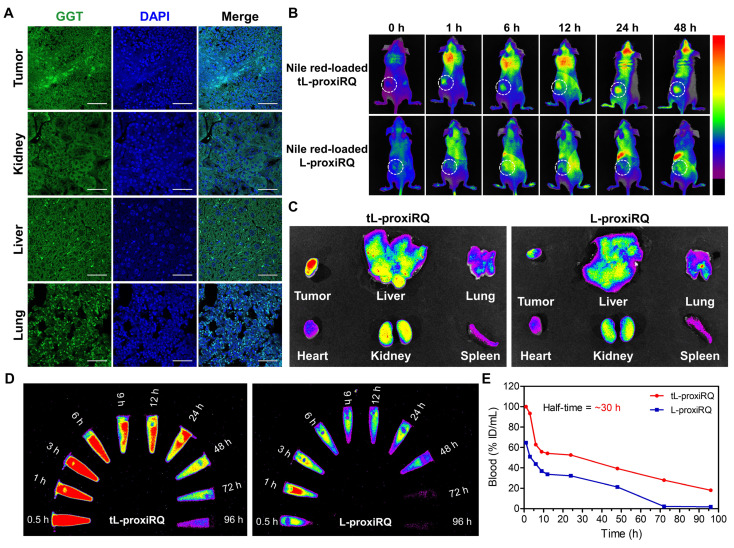
Biodistribution and blood pharmacokinetics of tL-proxiRQ. (A) Immunofluorescence analysis of GGT expression in the tumor and major organs. Scale bar is 50 µm. (B) Fluorescence images of tumor-bearing mice after injection of fluorescent tL-proxiRQ at different time points. (C) *Ex vivo* fluorescence images of tumors and major organs. (D) Fluorescence images of blood samples collected at indicated time intervals. (E) Quantitative analysis of blood fluorescence intensity after IR780-loaded liposomes injection.

GGT is widely known to be highly expressed in the kidneys and liver under physiological conditions, and is also overexpressed in certain tumor types, including MCF-7 breast cancer 48-50. To confirm tissue-specific GGT expression in our model, we performed immunofluorescence staining on kidneys, liver, lungs, and MCF-7 tumor tissues harvested from tumor-bearing mice. As shown in Figure 9A and S17, MCF-7 tumor tissue exhibited the most intense and punctate GGT-associated fluorescence, surpassing that of kidneys, liver and lungs, supporting elevated GGT expression in the tumor microenvironment. To evaluate the tumor-targeting ability of tL-proxiRQ that incorporates γPGA as a GGT-binding ligand, we intravenously injected Nile Red-loaded tL-proxiRQ or L-proxiRQ into MCF-7 tumor-bearing mice. Fluorescence imaging revealed a time-dependent tumor accumulation of tL-proxiRQ, with clearly detectable tumor-selective fluorescence as early as 6 h post-injection (Figure 9B). In contrast, L-proxiRQ displayed indiscernible tumor accumulation during the same time frame. *Ex vivo* imaging of excised organs supports these findings (Figure 9C and S18A). tL-proxiRQ showed the strongest fluorescence intensity in tumors, followed by the lungs, kidneys, and liver, achieving a remarkably high targeting efficiency of approximately 33%. In contrast, L-proxiRQ accumulated in the lungs and kidneys rather than the tumor, showing a lower efficiency of approximately 19%. The tumor-targeting efficiency was calculated as the percentage of fluorescence intensity per tumor weight relative to the sum of fluorescence intensities per weight of all excised organs (Figure S18B). These results indicate that the tumor accumulation of tL-proxiRQ is attributed not only to the enhanced permeability and retention (EPR) effect but also to active targeting *via* GGT recognition by γPGA.

IR780-loaded tL-proxiRQ was administered intravenously to rats at a dose of 20 mg/kg to investigate the pharmacokinetic profile. Blood samples were collected at predetermined time points and fluorescence imaging analysis was performed (Figure 9D-E). tL-proxiRQ exhibited a fluorescence half-life of approximately 30 h and maintained relatively stable fluorescence signals in circulation for up to 72 h. In contrast, the fluorescence intensity of L-proxiRQ decreased sharply immediately after injection and remained at marginal levels thereafter. Based on fluorescence intensity–time profiles, the AUC_0.5–96 h_ values of tL-proxiRQ and L-proxiRQ were estimated to be 429.1 and 200.9, respectively, which indicates that the systemic exposure of tL-proxiRQ increased by approximately 2-fold compared to L-proxiRQ.

Notably, a substantial contribution of the total AUC was observed for tL-proxiRQ in the 24–96 h period, suggesting that tL-proxiRQ exhibits improved and prolonged blood circulation behavior compared to L-proxiRQ. The prolonged circulation profile of tL-proxiRQ can be attributed to the introduction of the GGT-targeting γPGA ligand. γPGA is expected to enhance the colloidal stability of the liposomal formulation by imparting a negative charge, thereby reducing nonspecific protein adsorption and minimizing rapid clearance by mononuclear phagocytes. Collectively, the half-life and AUC analyses support the favorable systemic circulation properties of tL-proxiRQ, which is advantageous for tumor-targeted delivery.

### Antitumor activity of tL-proxiRQ in mouse xenograft models

**Figure 10:**
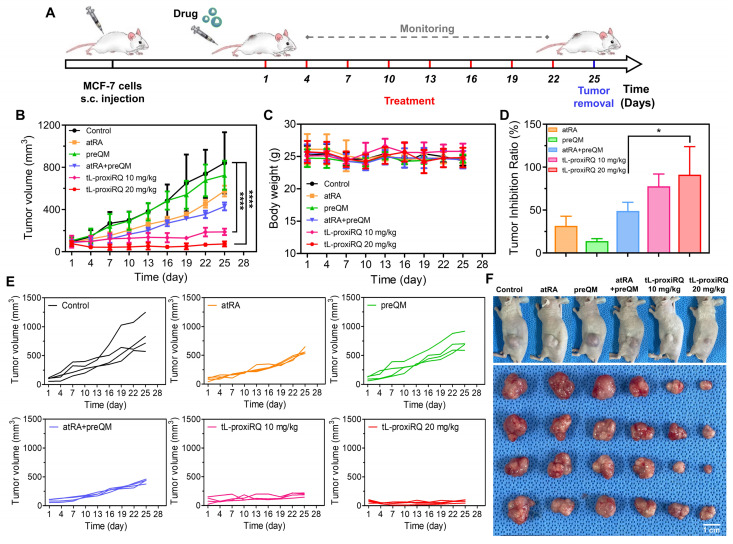
Antitumor activity of tL-proxiRQ. (A) Schematic experimental timeline for animal experiments. Mice were treated with various formulations every three days for 25 days. Changes in (B) tumor volume and (C) body weight were measured throughout the treatment period. (D) Tumor inhibition ratios relative to the control group. *P < 0.05. Values are mean ± s.d. (n = 4). (E) Individual tumor growth curves for each group. (F) Representative photographs of tumor-bearing mice and excised tumors.

To evaluate the *in vivo* antitumor activity of tL-proxiRQ, we established a xenograft model using MCF-7 human breast cancer cells. Mice were randomly assigned to six groups (n = 4 per group) and intravenously administered the following formulations: saline, atRA (3.7 mg/kg), preQM (2.9 mg/kg), a combination of atRA and preQM, and tL-proxiRQ (10 and 20 mg/kg). The administered doses of atRA and preQM were matched to their molar equivalents present in 20 mg/kg tL-proxiRQ. All formulations were administered every three days for a total of 25 days (Figure 10A). Tumor volume and body weight were measured at each dosing interval (Figure 10B-C). Body weight remained stable throughout the treatment period in all groups, indicating no apparent systemic toxicity. However, marked differences in tumor growth were observed among treatment groups. Tumors in the preQM-treated group grew comparably to those in the control (saline) group, suggesting limited antitumor activity. Administration of free atRA resulted in a slight delay in tumor growth. In particular, co-administration of atRA and preQM demonstrated an additive effect, with approximately 50% tumor growth inhibition compared to the control group (Figure 10D). In contrast, tL-proxiRQ exhibited a significant tumor suppression effect, ranging from 75% to 95%, at both 10 and 20 mg/kg doses. Individual tumor growth curves shown in Figure 10E, along with representative photographs of tumor-bearing mice and excised tumors, further demonstrated the superior antitumor effect of tL-proxiRQ (Figure 10F and S19). These findings suggest that tL-proxiRQ significantly enhances therapeutic efficacy through tumor-specific accumulation and the synchronous release of atRA and QM, thereby demonstrating potential as a potent and well-tolerated therapeutic system for breast cancer treatment.

**Figure 11:**
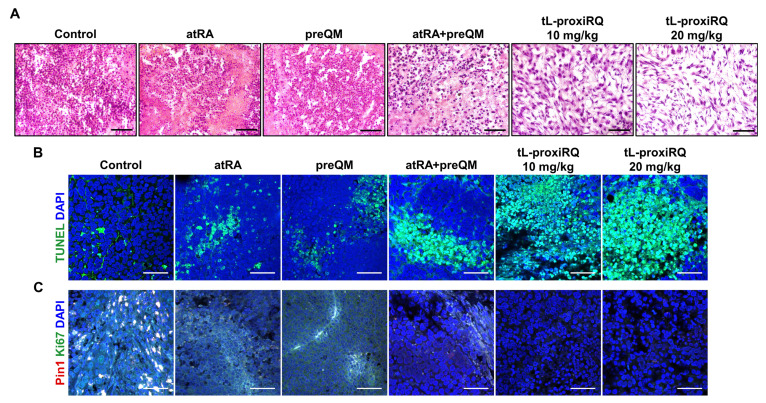
Histological examination of tumor tissues. (A) H&E-stained tumor tissues showing morphological alterations in tumor cells. Scale bar is 50 µm. (B) TUNEL-stained tumor tissues showing apoptotic cell death. Scale bar is 50 µm. (C) Immunofluorescence analysis of Pin1 and Ki-67 expression. Scale bar is 50 µm.

To further verify the therapeutic effect of tL-proxiRQ at the tissue level, histological and immunohistochemical analyses of excised tumor tissues were performed (Figure 11 and S20). H&E staining revealed pronounced morphological abnormalities in tumors of tL-proxiRQ-treated groups, including nuclear condensation, fragmentation, and cell membrane damage, suggesting apoptosis. Immunohistochemical analysis confirmed that apoptosis was promoted in the tL-proxiRQ-treated group, with a significant increase in TUNEL-positive cells. Concurrently, the expression of Ki-67, a cell proliferation marker, was markedly reduced, indicating that tumor cell growth was effectively inhibited. In addition, the expression level of Pin1, a key oncogenic regulator involved in tumor progression, was also significantly decreased in both tL-proxiRQ treated groups compared to the control group. These results support the rationale that tL-proxiRQ is activated by esterase within tumor cells, leading to simultaneous release of atRA and QM. The resulting dual pro-oxidant activity resulting from this release appears to contribute to redox imbalance, Pin1 degradation, and the induction of apoptosis. Collectively, the histological and immunohistochemical evidence corroborates the potent antitumor activity of tL-proxiRQ observed *in vivo*, underscoring a mechanism involving intracellular esterase-responsive activation and dual-pathway cytotoxicity.

### Biosafety evaluation of tL-proxiRQ

**Figure 12:**
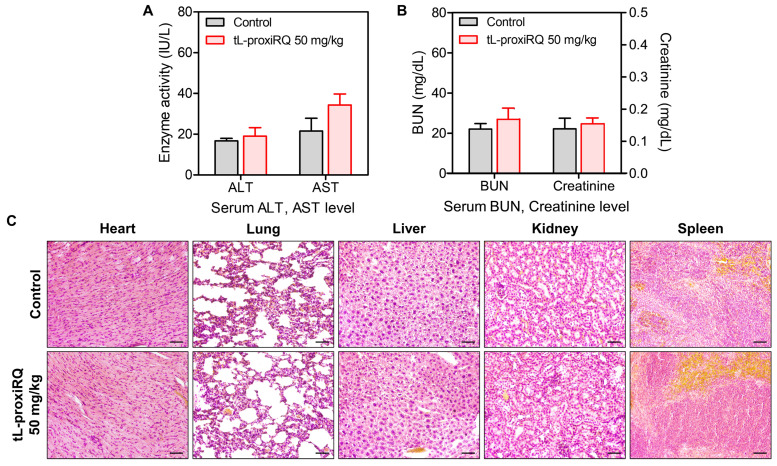
Biosafety evaluation of tL-proxiRQ. Serum levels of (A) ALT, AST, (B) BUN, and Cr in mice treated with tL-proxiRQ and control. Values are mean ± s.d. (n = 3). (C) H&E-stained sections of major organs. Scale bar is 50 µm.

The *in vivo* safety profile of tL-proxiRQ was assessed by intravenously administering 50 mg/kg, equivalent to 2.5 times the therapeutic dose used in tumor models, on alternate days over 10 days to healthy mice. After administration, blood samples and major organs, including the hearts, lungs, liver, kidneys, and spleen, were collected for serum biochemical and histological analysis. Serum ALT, AST, BUN, and Cr levels were slightly elevated but remained within the normal physiological range, suggesting no significant hepatic and renal toxicity (Figure 12A-B). Moreover, histological examination of H&E-stained tissues revealed no pathological abnormalities in any of the examined organs (Figure 12C). These results demonstrate that tL-proxiRQ exhibits a favorable safety profile even at supratherapeutic doses, supporting its potential for future preclinical development and clinical applications.

## Discussion

atRA and QM have attracted increasing attention as potential anticancer agents owing to their ability to modulate oxidative stress and suppress oncogenic signaling. In cell culture studies, we observed a significant synergistic effect when atRA and the QM precursor were combined at a 1:1 molar ratio. However, this synergy is difficult to maintain *in vivo* because the distinct and unfavorable pharmacokinetic profiles of free atRA and QM lead to asynchronous tumor exposure and limited intracellular co-availability. Therefore, to fully realize their therapeutic synergy *in vivo*, it is essential to develop a delivery strategy capable of simultaneously releasing both agents intracellularly in a spatiotemporally controlled manner. To address this challenge, we developed proxiRQ as a self-synergizing mutual prodrug, in which the atRA and QM precursor are integrated into a single molecular scaffold containing an esterase-cleavable bond. When proxiRQ is enzymatically activated within cancer cells, it coordinately releases both active components.

**Scheme 1:**
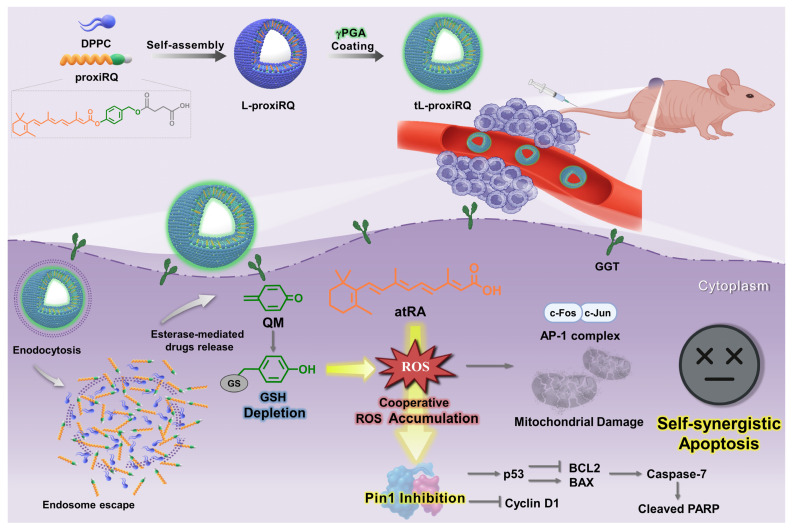
Schematic illustration of targeted cancer therapy using tL-proxiRQ, a self-synergizing mutual prodrug liposomal nanomedicine. tL-proxiRQ targets cancer cells through GGT-recognition and synchronously delivers both atRA and QM, which amplify ROS accumulation and induce Pin1 inhibition, thereby achieving synergistic anticancer efficacy.

As shown in Scheme 1, the conjugation of the carboxylated QM precursor to the carboxylic acid of atRA elongates the molecular length and imparts distinct amphiphilicity. This structural modification confers self-assembly capability, facilitating efficient and cooperative integration into a lipid bilayer. When co-formulated with DPPC, proxiRQ assembled into a stable liposomal structure (tL-proxiRQ) with an exceptionally high drug loading of approximately 37 mol%, reflecting favorable molecular alignment with phospholipid components. Notably, because cholesterol and proxiRQ sterically compete for the hydrophobic core of the lipid bilayer, we maximized drug loading capacity by designing cholesterol-free liposomes. Intriguingly, despite the absence of cholesterol, tL-proxiRQ exhibited excellent colloidal stability for over 30 days without aggregation or changes in morphology. These results suggest that the rigid polyene tail of proxiRQ can partially compensate for the membrane-stabilizing role performed by cholesterol, thereby maintaining the structural stability of the bilayer while simultaneously serving as the therapeutic payload. This dual functionality distinguishes proxiRQ from conventional prodrugs and enables streamlined formulation, high drug content, and a scalable platform that is well-suited for further translational development.

tL-proxiRQ induces a dual ROS-based anticancer effect by undergoing esterase-mediated cleavage to spatiotemporally release atRA and QM. The atRA-induced ROS generation within cancer cells is further amplified by intracellular GSH depletion caused by QM, which ultimately leads to a severe disturbance of redox homeostasis. This extreme imbalance causes mitochondrial dysfunction and regulates the expression of ROS-responsive genes (Figure 6 and 8A). Furthermore, the amplified oxidative stress triggers the oxidation of highly reactive cysteine residues (particularly Cys113) within the Pin1 catalytic site, which impairs the intrinsic isomerase activity of Pin1 and induces structural instability. This leads to proteasome-mediated degradation, resulting in reduced Pin1 protein expression (Figure 2F-G and 8C). In addition to the well-known direct Pin1-inhibitory effect of atRA, this series of oxidative processes leads to the potent inactivation of Pin1 and the subsequent activation of apoptotic pathways. Consistent with these cellular-level mechanistic insights, the clear therapeutic benefits of tL-proxiRQ were further demonstrated *in vivo* experiments. γPGA, introduced for GGT-mediated tumor targeting, unexpectedly improved pharmacokinetic properties and prolonged the systemic circulation time (Figure 9B-E). Compared with the free atRA, preQM, or their combination treatment, tL-proxiRQ achieved significantly enhanced tumor suppression at doses lower than those typically used in preclinical studies of drug-loaded nanoparticles, without distinct systemic toxicity 51, 52. This therapeutic benefit can be explained by the high drug loading capacity (37 mol%), γPGA -mediated tumor targeting, and synchronized intracellular release of therapeutic agents. These characteristics also overcome the pharmacokinetic limitations that often constrain conventional combination therapies.

While these findings demonstrate the promising clinical potential of tL-proxiRQ, several considerations warrant further investigation prior to clinical translation. Although γPGA-mediated targeting effectively enhances tumor accumulation, uptake to extra-tumor sites may occur because GGT is expressed in stromal components such as tumor-associated macrophages and endothelial cells. While this extensive intratumoral distribution might offer some therapeutic advantages, further refinement of target specificity could improve safety and efficacy. Additionally, although the biological safety profile observed in this study appears favorable, long-term toxicity, immunogenicity, and applicability to various tumor types require thorough investigation. Although we confirmed the downregulation of Pin1-dependent metastatic markers, such as Cyclin D1 and MMP-9, and observed reduced wound closure in the wound healing assay (Figure S21), more in-depth verification is required regarding the anti-metastatic potential of tL-proxiRQ. Future studies employing *in vivo* spontaneous metastasis models should fully elucidate the efficacy of our strategy for inhibiting systemic cancer spread. From a translational perspective, further optimization of dosing regimens, including dose escalation and frequency studies, will be essential to define the optimal therapeutic range and ensure maximum efficacy with minimal toxicity. Furthermore, direct comparison with clinically used chemotherapeutic agents or standard combination regimens would help contextualize the therapeutic benefits. Finally, it should be noted that atRA can undergo photo- and thermal isomerization into 9- or 13-*cis* forms under physiological conditions, and that this process is generally reversible and controllable through appropriate storage and formulation 53, 54. proxiRQ was synthesized using atRA, and although isomerization was expected to be minimized due to covalent linkage within this mutual prodrug structure, no isomerization-related effects were observed in this study. Nevertheless, further investigation into the stability and biological activity of potential isomer forms would be valuable for ensuring the robustness and translational reliability of the proxiRQ platform.

## Conclusion

In conclusion, this study establishes tL-proxiRQ as a novel liposomal nanomedicine built upon a self-synergizing mutual prodrug strategy that integrates therapeutic function with structural utility. By covalently linking atRA and a QM precursor *via* an esterase-cleavable bond, proxiRQ achieves synchronized intracellular release of both agents, thereby amplifying redox stress and suppressing the oncogenic regulator Pin1 through complementary and convergent mechanisms. Importantly, this molecular integration addresses a fundamental limitation of conventional combination therapy by embedding synergy directly into the drug architecture rather than relying on co-administration. The amphiphilic nature of proxiRQ not only allows an exceptionally high drug loading (37 mol%) but also reinforces membrane stability, supporting the formation of cholesterol-free, structurally robust liposomes. The resulting formulation, tL-proxiRQ, demonstrates superior anticancer efficacy compared with the free drug combination, underscoring the therapeutic advantage of synchronized delivery and dual-mechanism action. Additionally, the incorporation of γPGA, which was originally introduced for GGT-mediated tumor targeting, unexpectedly improved systemic circulation, collectively overcoming key pharmacokinetic and biodistribution challenges that often limit combination chemotherapy. Taken together, these findings establish tL-proxiRQ as a promising nanomedicine platform with high translational potential for targeting aggressive cancer cells. More broadly, this study highlights mutual prodrug-driven self-assembling nanostructures as a generalizable framework for the rational design of next-generation combination nanomedicines, offering a streamlined and mechanistically integrated therapeutic paradigm.

## Supplementary Material

Supplementary figures and tables.

## Data Availability

The data supporting the results of this study will be provided by the corresponding authors upon a reasonable request.

## Abbreviations

atRA: all-*trans* retinoic acid
QM: quinone methide
ROS: reactive oxygen species
DPPC: dipalmitoyl phosphatidylcholine
γPGA: poly(γ-glutamic acid)
GSH: glutathione
Pin1: peptidyl-prolyl *cis-trans* isomerase NIMA-interacting 1
RARα: retinoic acid receptor alpha
GGT: γ-glutamyl transferase
THF: tetrahydrofuran
DCM: dichloromethane
HBA: 4-hydroxybenzyl alcohol
DMF: dimethylformamide
DIPEA: *N*,*N*-diisopropylethylamine
DMAP: 4-(dimethylamino)pyridine
EtOAc: ethyl acetate
PBS: phosphate-buffered saline
TEM: transmission electron microscopy
FBS: fetal bovine serum
DLC: drug loading capacity
EE: encapsulation efficiency
DCFH-DA: 2′,7′-dichlorofluorescin diacetate
ΔΨ_m_: mitochondrial membrane potential
FITC: fluorescein isothiocyanate
PI: propidium iodide
FSC: forward scatter
SSC: side scatter
DMSO: dimethyl sulfoxide
MTT: 3-(4,5-dimethylthiazol-2-yl)-2,5-diphenyltetrazolium bromide
NAC: *N*-acetyl-L-cysteine
qPCR: quantitative polymerase chain reaction
cDNA: complementary DNA
TBST: Tris-buffered saline containing 0.1% Tween-20
AUC: area under the curve
ALT: alanine transaminase
AST: aspartate transaminase
BUN: blood urea nitrogen
Cr: creatinine
H&E: hematoxylin and eosin
s.d.: standard deviation
preQM: QM precursor
CI: combination index
Fa: fractional affected
DLS: dynamic light scattering
HO-1: heme oxygenase-1
TUNEL: terminal deoxynucleotidyl transferase dUTP nick end labeling
