# Supplementary material for: Self-synergizing mutual prodrug liposomes for targeted cancer therapy via redox-amplified Pin1 inhibition
Source: Theranostics. 2026 Jun 10;16(13):7442–65. doi: 10.7150/thno.131597 (PMC13295116; doi:10.7150/thno.131597)
Supplement: Supplementary file 1 — Supplementary figures and tables. [file thnov16p7442s1.pdf]

Supplementary information

Self-synergizing mutual prodrug liposomes for targeted cancer therapy *via* redox-amplified Pin1 inhibition-

Nuri Kim [a], Ilseob Kim [b], Hanui Jo [a], Nanhee Song [a], Sangmin Jo [a], Suyeon Lee [a], Hoechang Kim [a], Changjin Lim [b,*], Dongwon Lee [a,c,*]

[a] Department of Bionanotechnology and Bioconvergence Engineering, Jeonbuk National University, Jeonju, Jeonbuk, 54896, Republic of Korea

[b] Department of Pharmacy, Jeonbuk National University, Jeonju, Jeonbuk, 54896, Republic of Korea

[c] Department of Polymer· Nano Science and Technology, Jeonbuk National University, Jeonju, Jeonbuk, 54896, Republic of Korea

Corresponding author: Dongwon Lee, E-mail: dlee@jbnu.ac.kr

Keywords: all-trans retinoic acid; prodrug; cancer; liposome; Pin1; redox homeostasis

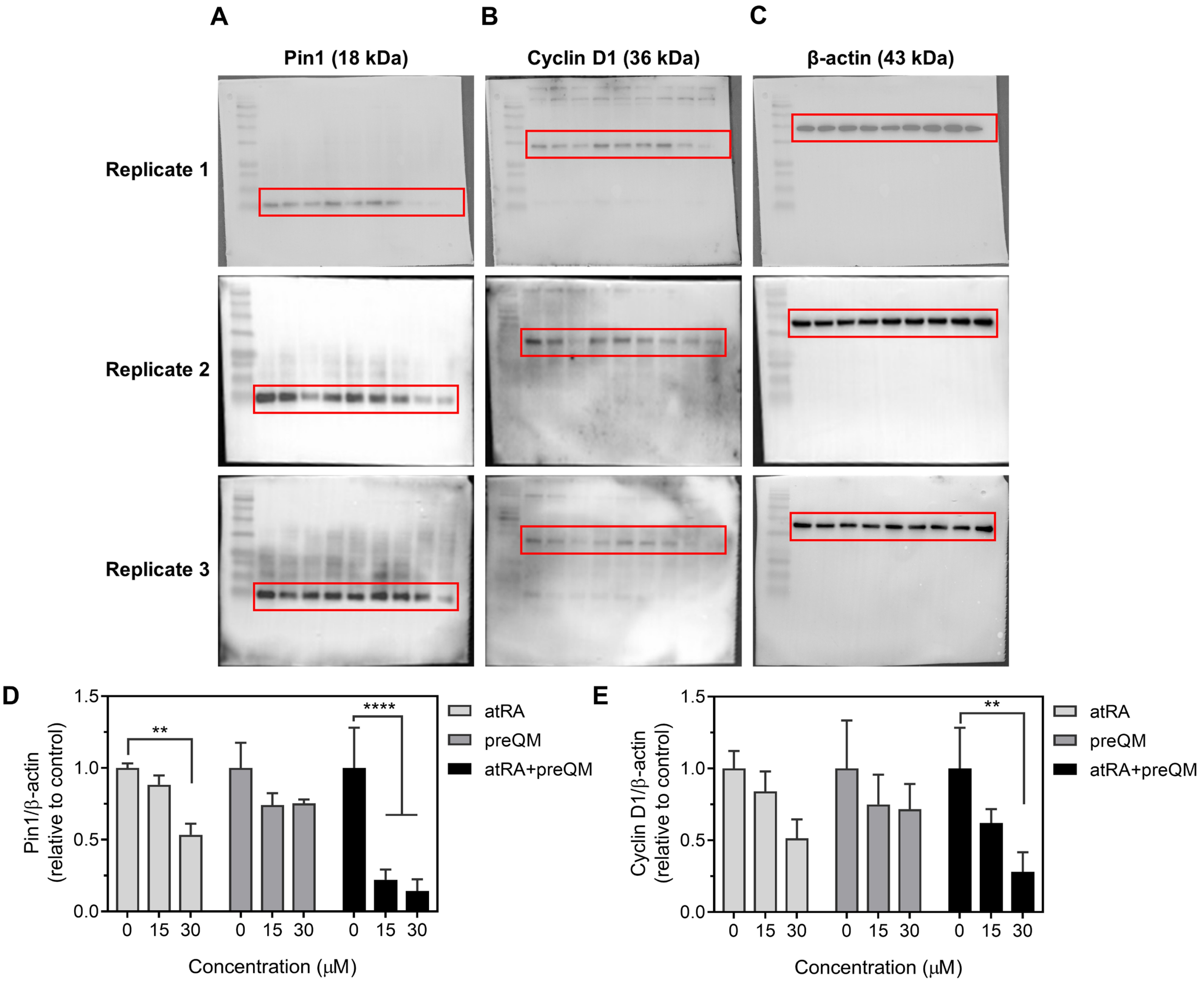

Figure S1. Uncropped Western blot images and quantitative analysis corresponding to Figure 1C. Uncropped Western blot images showing the expression of (A) Pin1 and (B) Cyclin D1 in MCF-7 cells. (C) β-actin was used as a loading control. The red box indicates the regions presented in the main figure. Relative protein expression levels of (D) Pin1 and (E) Cyclin D1 were quantified by densitometry and normalized to β-actin. $^{**}P < 0.01$, $^{****}P < 0.0001$ relative to the untreated group. Values are mean ± s.d. (n = 3).

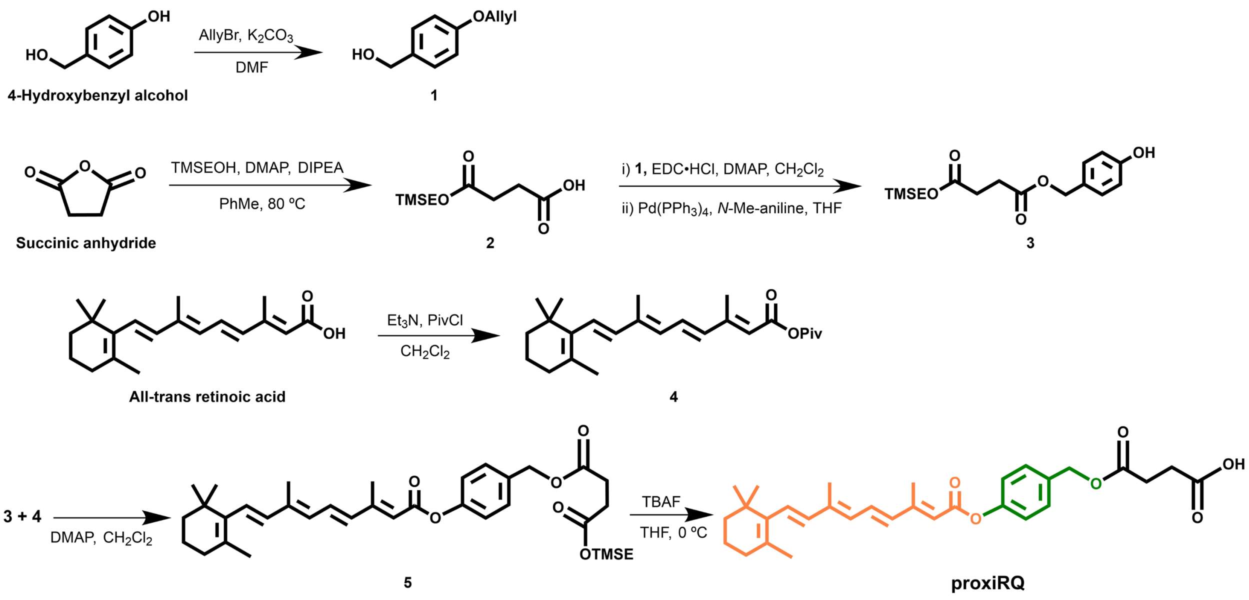

Figure S2. Synthetic route for proxiRQ.

A

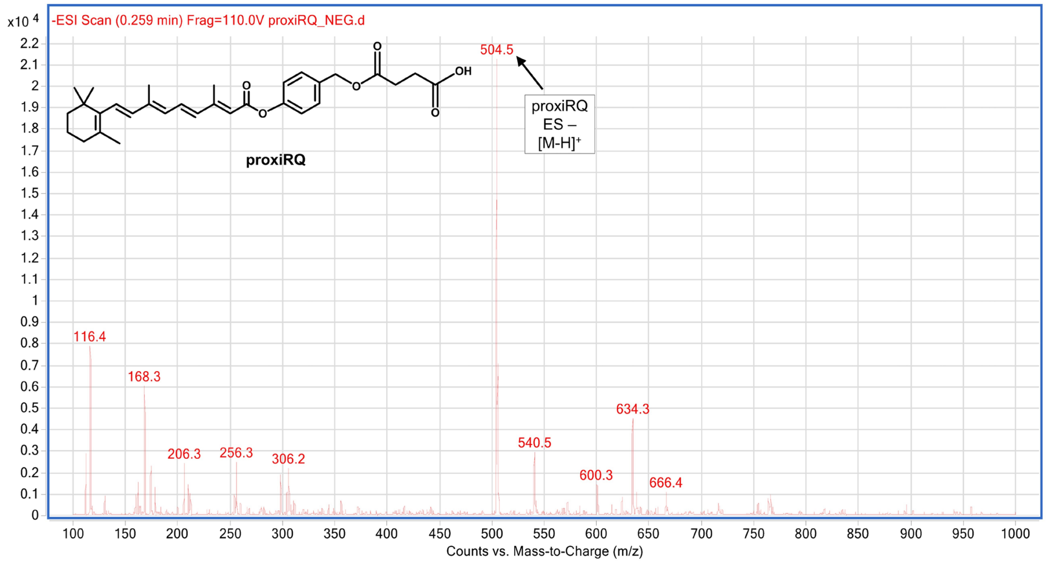

B

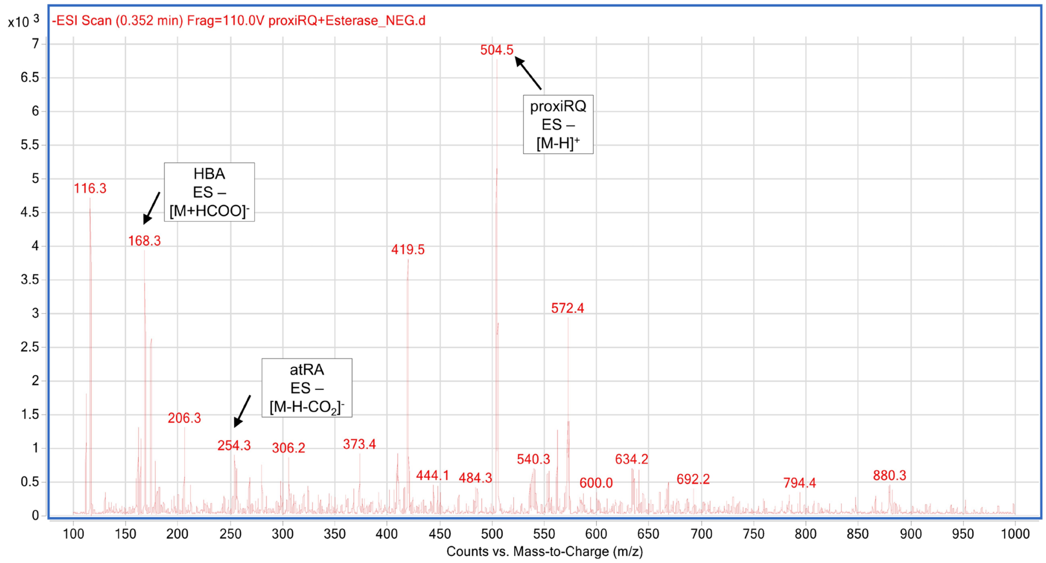

Figure S3. LC-MS/MS analysis of proxiRQ degradation by esterase. Representative LC-MS/MS spectra of proxiRQ (A) in the absence of esterase and (B) after incubation with esterase. The presence of characteristic peaks in (B) demonstrates the enzymatic release of atRA and HBA from the proxiRQ.

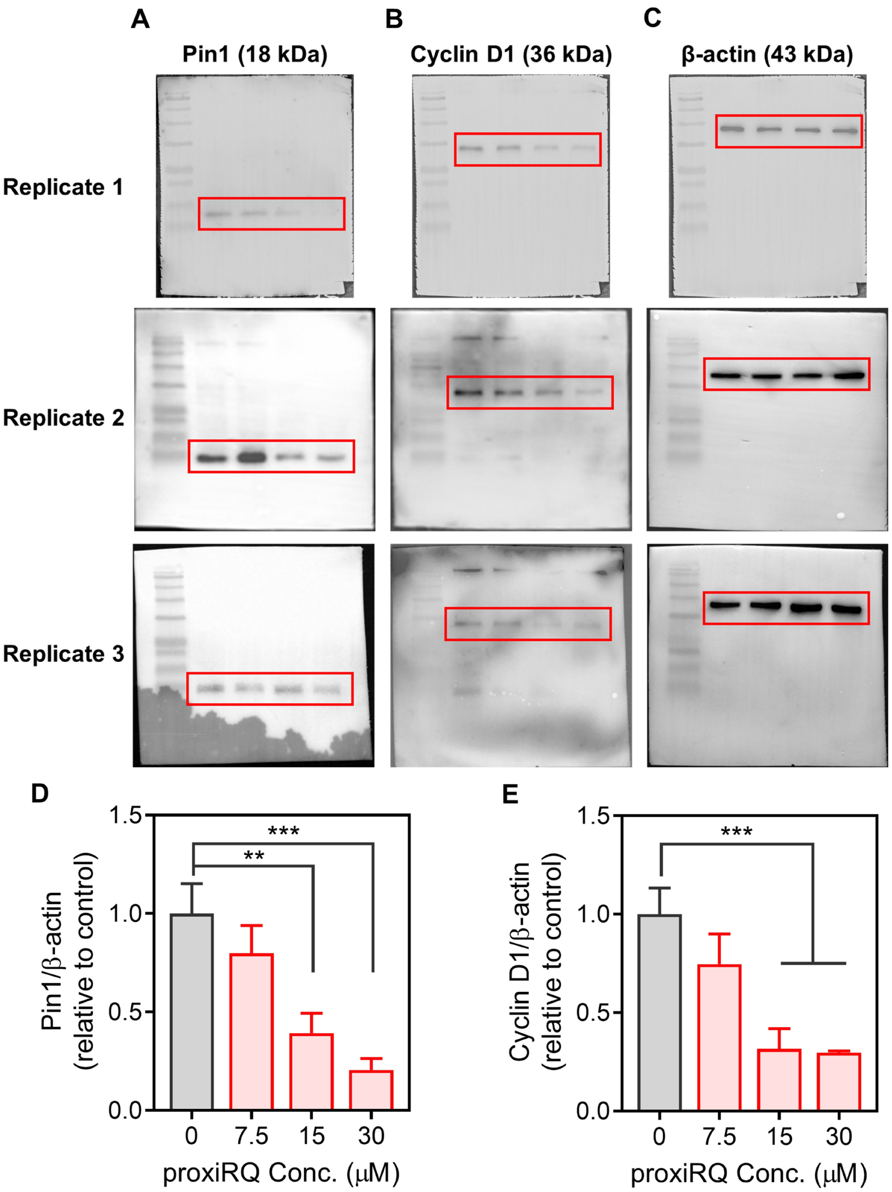

Figure S4. Uncropped Western blot images and quantitative analysis corresponding to Figure 2G. Uncropped Western blot images showing the expression of (A) Pin1 and (B) Cyclin D1 in MCF-7 cells. (C) β-actin was used as a loading control. The red box indicates the regions presented in the main figure. Relative protein expression levels of (D) Pin1 and (E) Cyclin D1 were quantified by densitometry and normalized to β-actin. $^{**}P < 0.01$, $^{***}P < 0.001$ relative to the untreated group. Values are mean ± s.d. (n = 3).

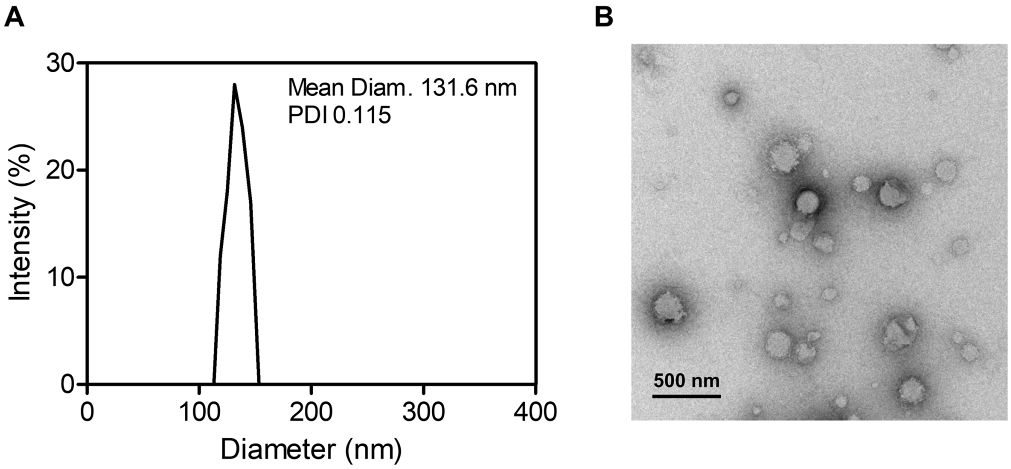

Figure S5. Characterization of L-proxiRQ. (A) Size distribution and (B) TEM image.

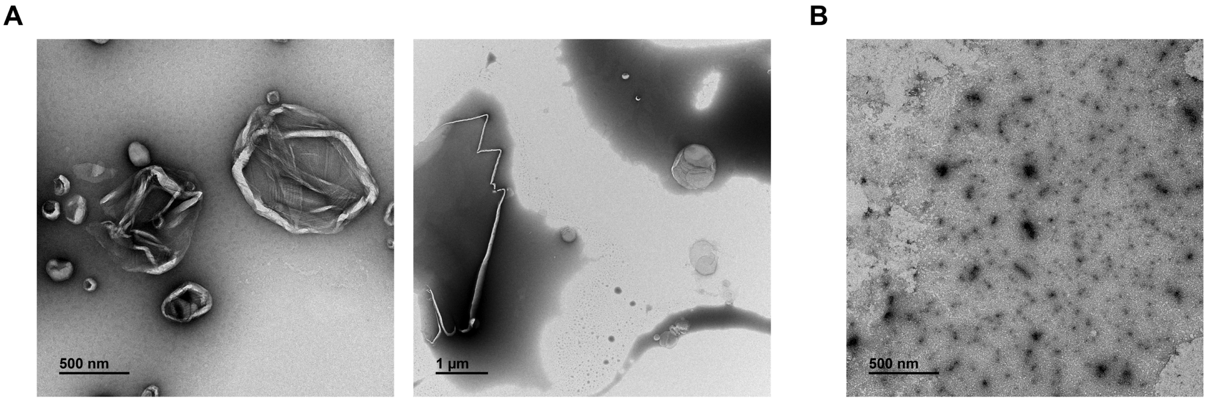

Figure S6. TEM images showing the failure of liposome formation. Liposomes were prepared with (A) 60 mol% DPPC and 40 mol% atRA, and (B) 60 mol% DOTAP and 40 mol% proxiRQ. No vesicular structures were observed under either condition.

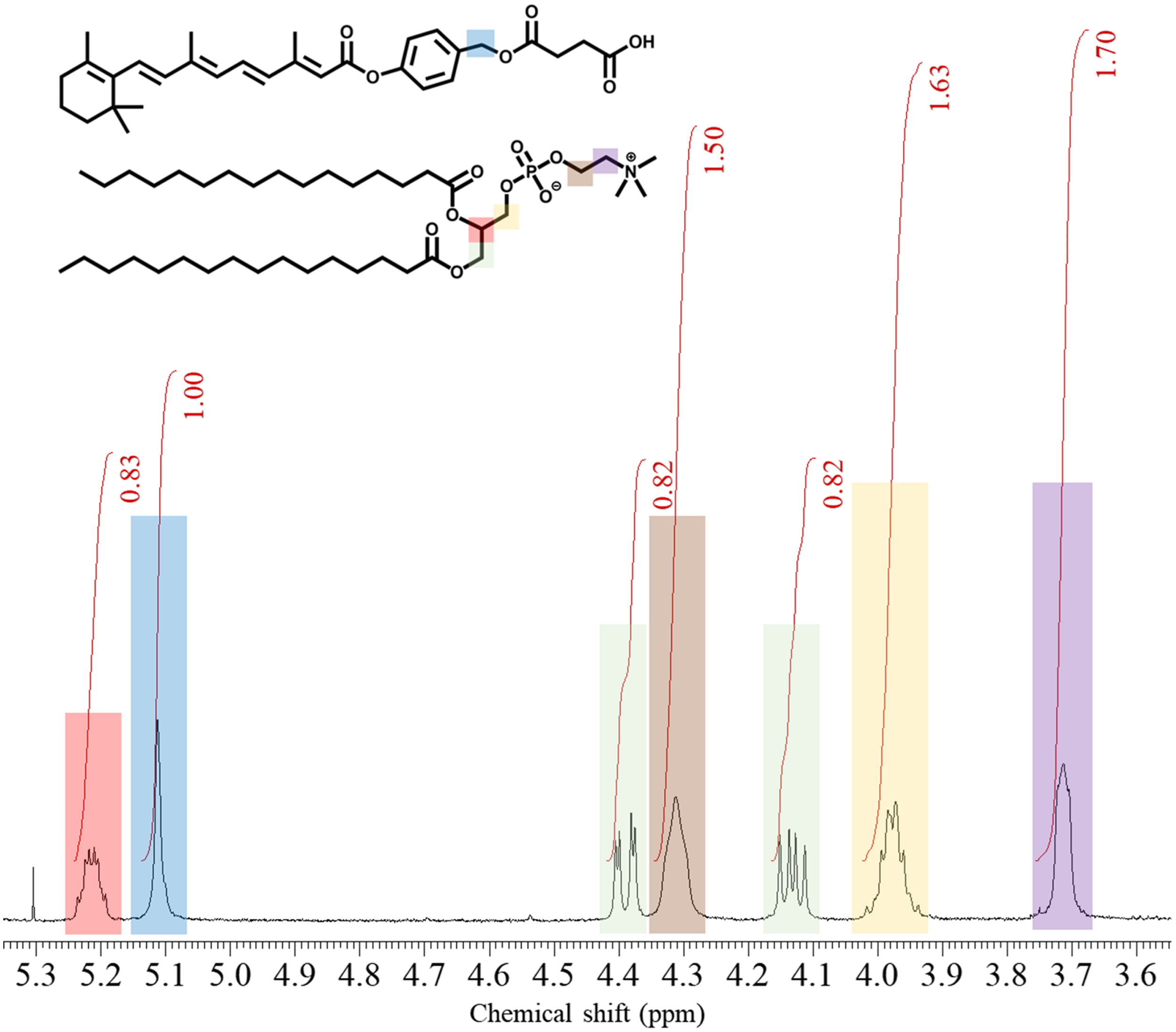

Figure S7. Quantification of proxiRQ content in L-proxiRQ by ^{1}H NMR. Lyophilized tL-proxiRQ powder was dissolved in $CDCl_3$ and analyzed by ^{1}H NMR. The integrals of DPPC- and proxiRQ-specific proton resonances were compared to determine the compositional ratio of the two components in the final formulation. Peaks corresponding to each component are highlighted in distinct colors for clarity.

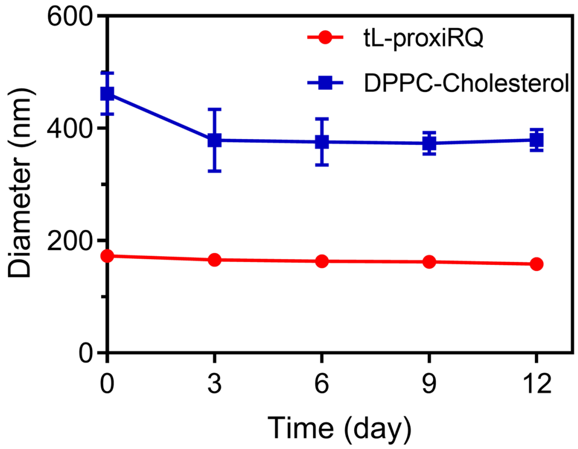

Figure S8. Long-term colloidal stability of tL-proxiRQ and control liposomes. Physical stability was evaluated by measuring the hydrodynamic size of tL-proxiRQ (DPPC/proxiRQ, 6:4 molar ratio) and standard control liposomes (DPPC/cholesterol, 6:4 molar ratio) using DLS. The particle size was measured at 3-day intervals for 12 days. Values are mean ± s.d. (n = 3).

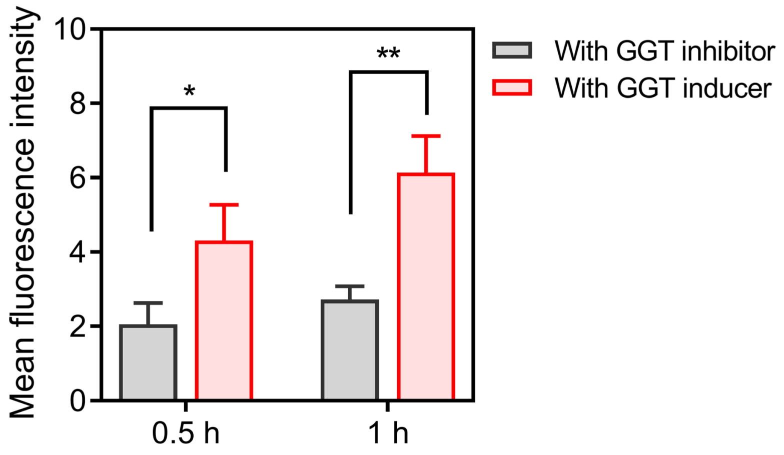

Figure S9. Quantitative analysis of GGT-dependent cellular uptake of tL-proxiRQ. Fluorescence intensities were quantified from confocal microscopy images of MCF-7 cells under GGT-inhibited or GGT-induced conditions. The mean fluorescence intensity was calculated using ImageJ software. $^{*}P < 0.05$, $^{**}P < 0.01$ (Student's *t*-test). Values are mean ± s.d. (n = 4).

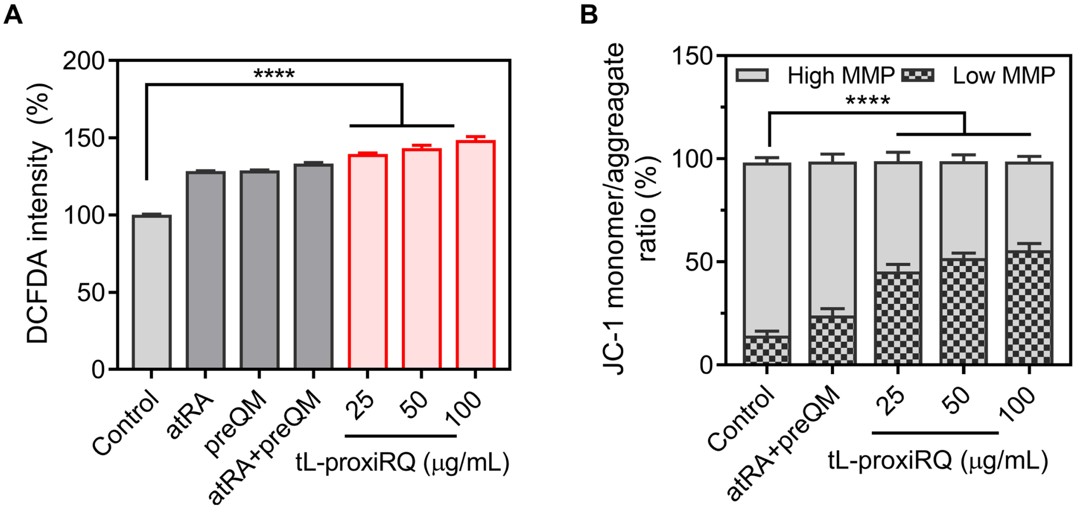

Figure S10. Quantitative analysis of intracellular ROS levels and mitochondrial membrane potential. (A) Relative DCFDA fluorescence intensities were quantified from confocal microscopy images of MCF-7 cells. The mean fluorescence intensity was calculated using ImageJ software. $^{****}P < 0.0001$ relative to the control. Values are mean ± s.d. (n = 4). (B) Mitochondrial damage was assessed *via* FACS after JC-1 staining. $^{****}P < 0.0001$ relative to the control. Values are mean ± s.d. (n = 3).

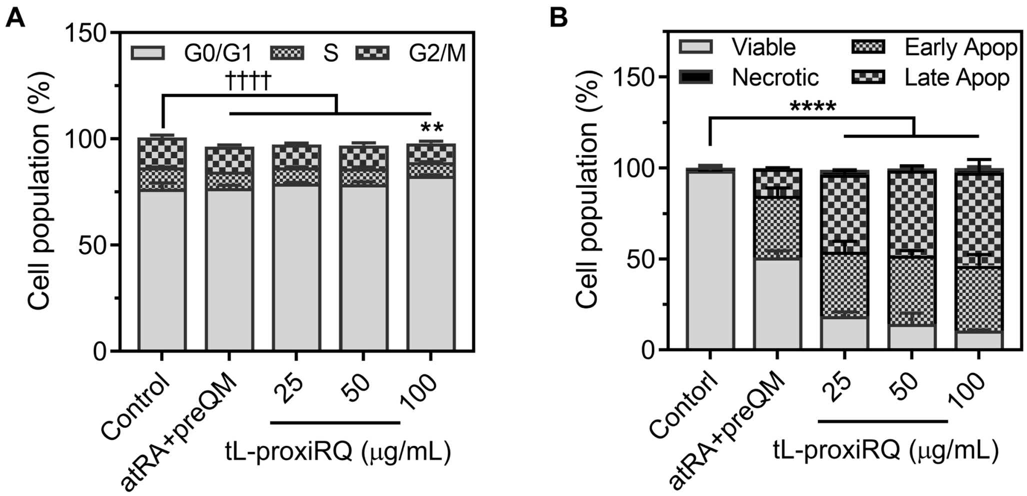

Figure S11. Quantitative flow cytometry analysis. (A) Cell cycle arrest was analyzed by PI staining in MCF-7 cells. The percentage of cells in the G0/G1, S, and G2/M phases was determined using the instrument's analysis software. $^{**}P < 0.01$ relative to the control for the G0/G1 phase; $^{††††}P < 0.0001$ relative to the control for the S phase. Values are mean ± s.d. (n = 3). (B) Apoptosis induction was evaluated by Annexin V/PI dual staining. Cells were classified into four populations: viable, early-apoptotic, late-apoptotic, and necrotic cells. The percentage of each population was calculated from the FACS data. $^{****}P < 0.0001$ relative to the control. Values are mean ± s.d. (n = 3).

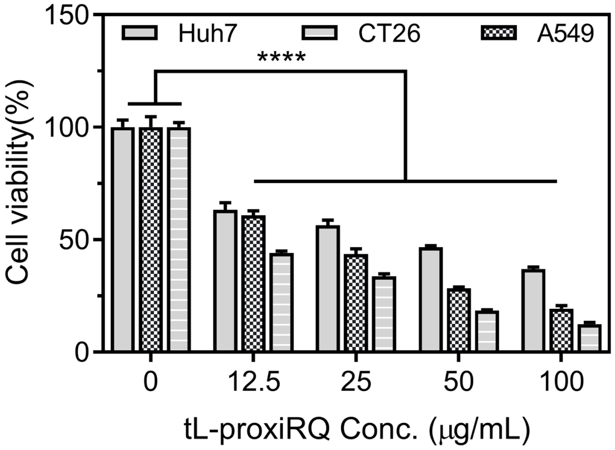

Figure S12. Cytotoxicity of tL-proxiRQ in various cancer cell lines. To evaluate the broad applicability of tL-proxiRQ, MTT assays were performed in Huh7, A549, and CT26 cells. $^{****}P < 0.0001$ relative to the control. Values are mean ± s.d. (n = 4).

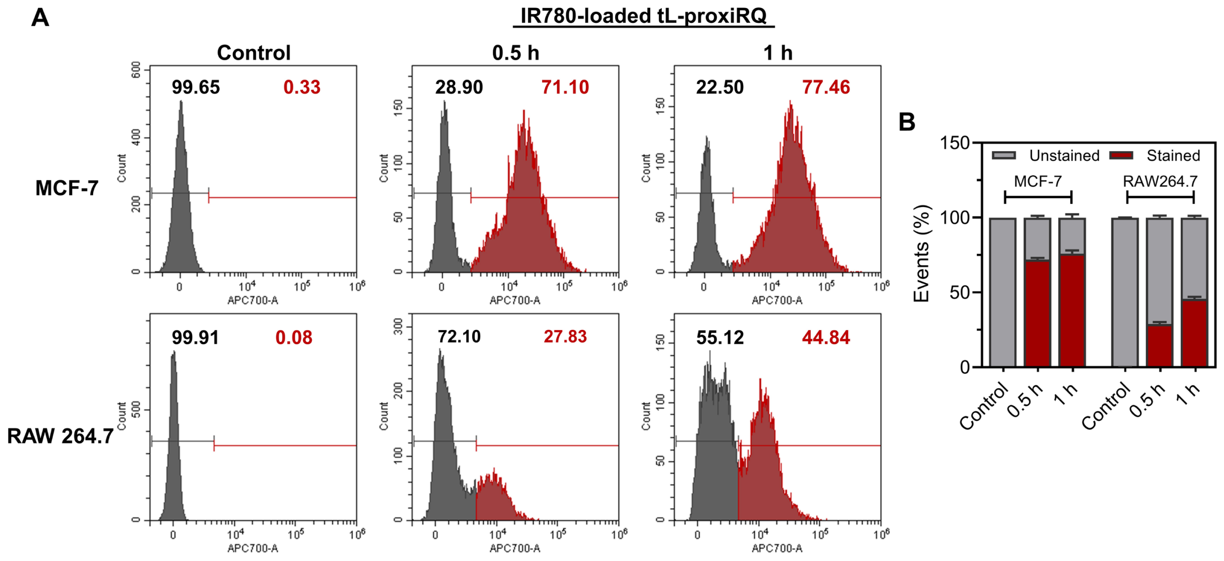

Figure S13. Comparative analysis of cellular uptake of tL-proxiRQ in MCF-7 and RAW 264.7 cells. (A) Representative FACS histograms showing the cellular uptake of IR780-loaded tL-proxiRQ after 0.5 and 1 h of incubation. (B) Quantitative analysis of the mean fluorescence intensity. The uptake efficiency and rate between the two cell lines were compared at each time point. Values are mean ± s.d. (n = 3).

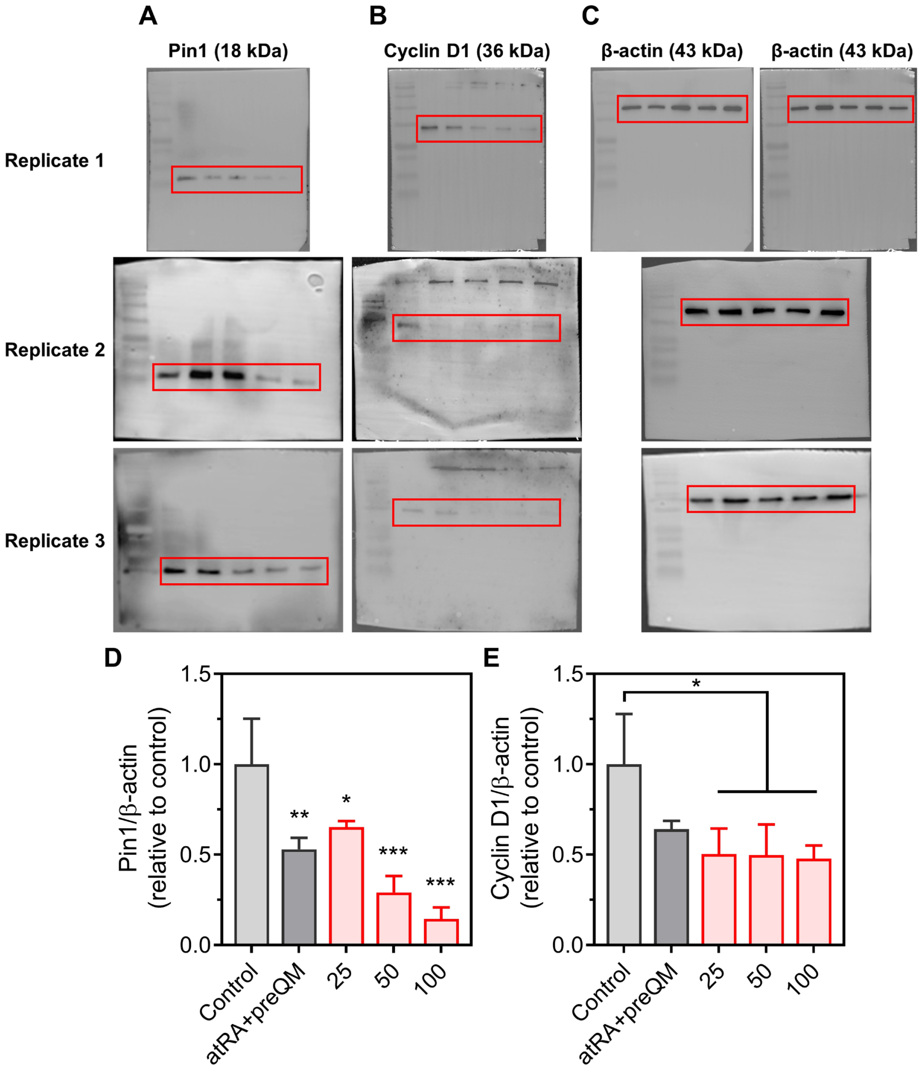

Figure S14. Uncropped Western blot images and quantitative analysis corresponding to Figure 8C, showing the expression of (A) Pin1 and (B) Cyclin D1 in MCF-7 cells. (C) β-actin was used as a loading control. The red box indicates the regions presented in the main figure. Relative protein expression levels of (D) Pin1 and (E) Cyclin D1 were quantified by densitometry and normalized to β-actin. $^{*}P < 0.05$, $^{**}P < 0.01$, $^{***}P < 0.001$ relative to the control. Values are mean ± s.d. (n = 3).

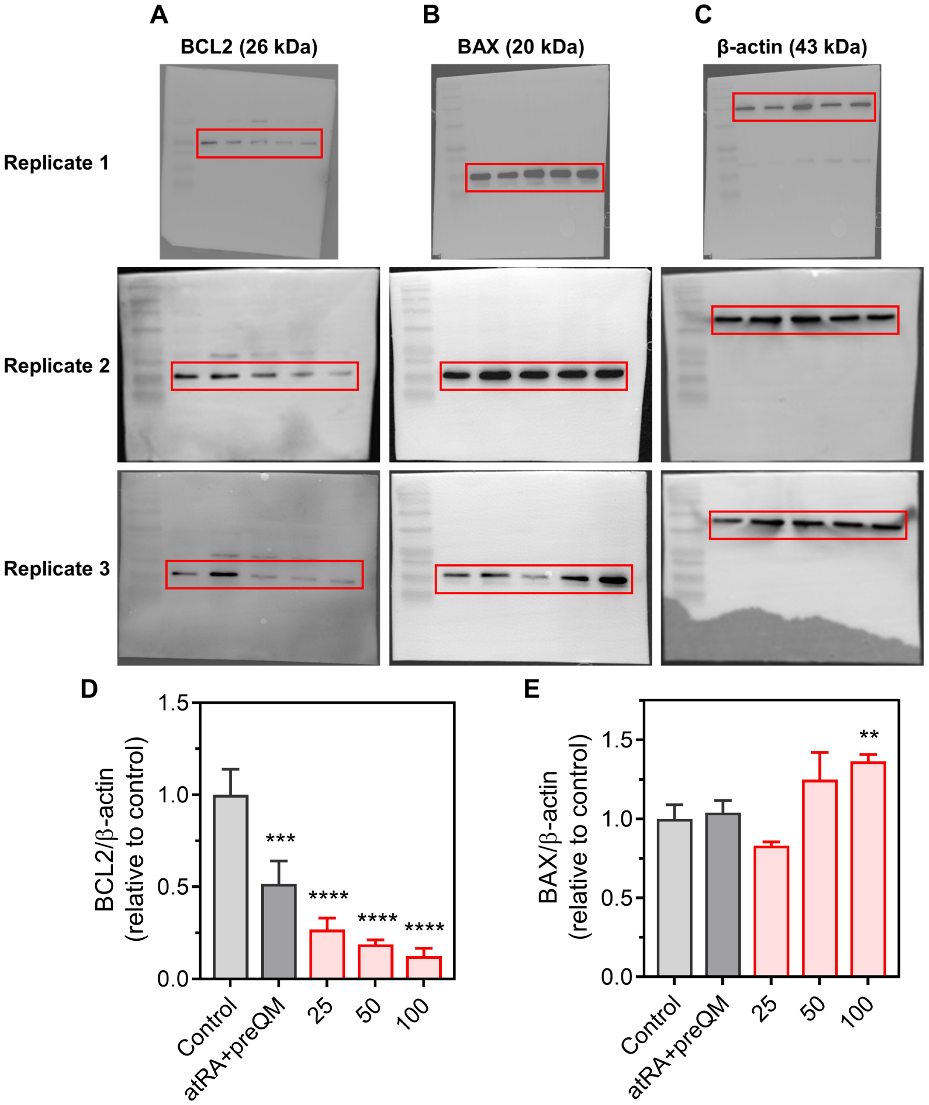

Figure S15. Uncropped Western blot images and quantitative analysis corresponding to Figure 8D. Uncropped Western blot images showing the expression of (A) BCL-2 and (B) BAX in MCF-7 cells. (C) β-actin was used as a loading control. The red box indicates the regions presented in the main figure. Relative protein expression levels of (D) BCL-2 and (E) BAX were quantified by densitometry and normalized to β-actin. $^{**}P < 0.01$, $^{***}P < 0.001$, $^{****}P < 0.0001$ relative to the control. Values are mean ± s.d. (n = 3).

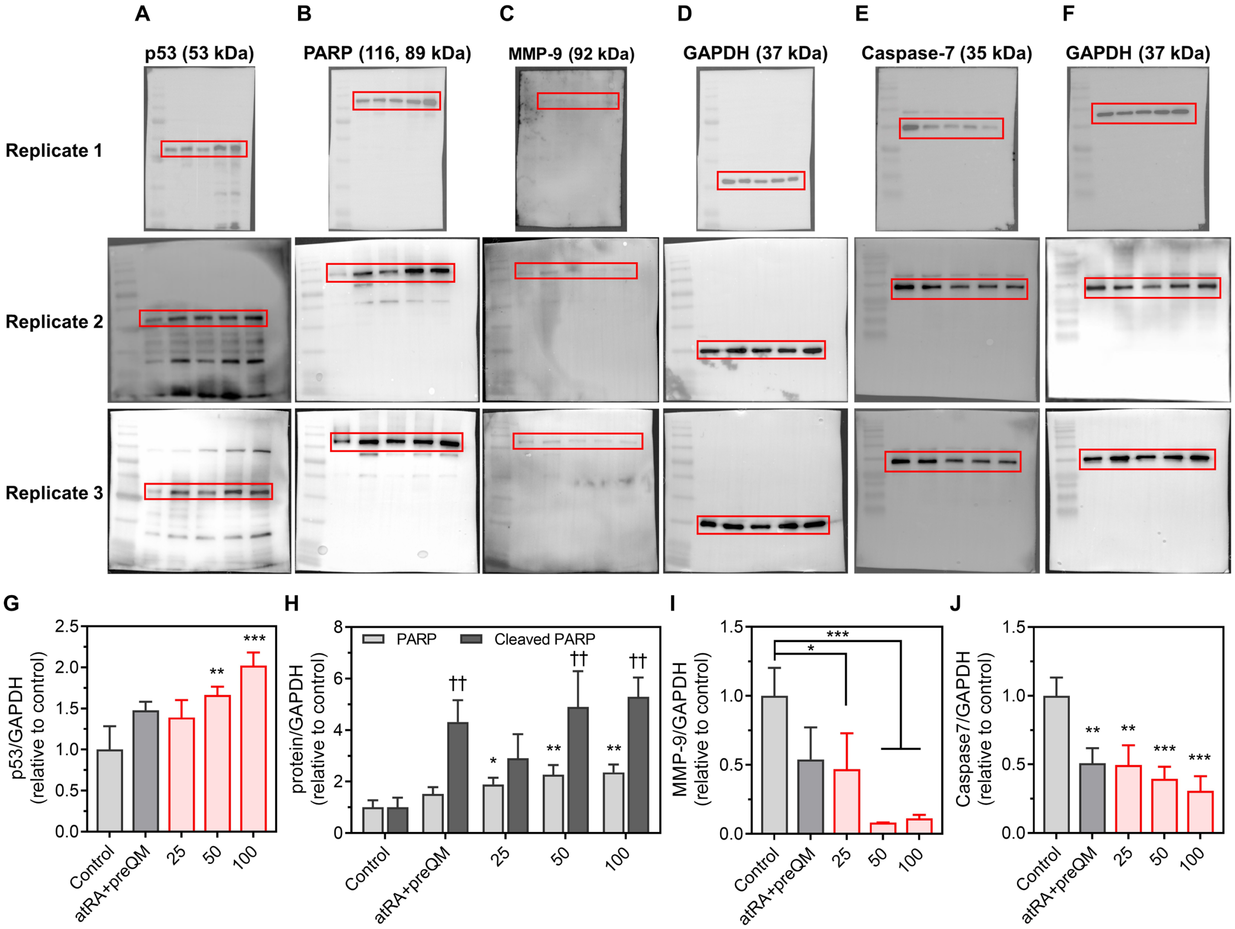

Figure S16. Uncropped Western blot images and quantitative analysis corresponding to Figure 8C and E, showing the expression of (A) p53, (B) PARP, cleaved PARP, (C) MMP-9, and (E) Caspase-7 in MCF-7 cells. Panels (D) and (F) show GAPDH, which was used as a loading control. The red box indicates the regions presented in the main figure. Relative protein expression levels of (G) p53, (H) PARP, cleaved PARP, (I) MMP-9, and (J) Caspase-7 were quantified by densitometry and normalized to GAPDH. $^{*}P < 0.05$, $^{**}P < 0.01$, $^{***}P < 0.001$ relative to the control. $^{\dagger\dagger}P < 0.01$ relative to the control. Values are mean ± s.d. (n = 3).

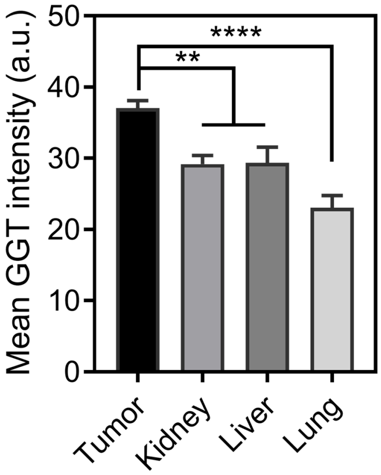

Figure S17. Quantitative analysis of GGT expression in the tumor and major organs corresponding to Figure 9a. Relative GGT expression levels were quantified from confocal immunofluorescence images using imageJ software. $^{**}P < 0.01$, $^{****}P < 0.0001$ relative to the mean GGT intensity of the tumor. Values are mean ± s.d. (n = 4).

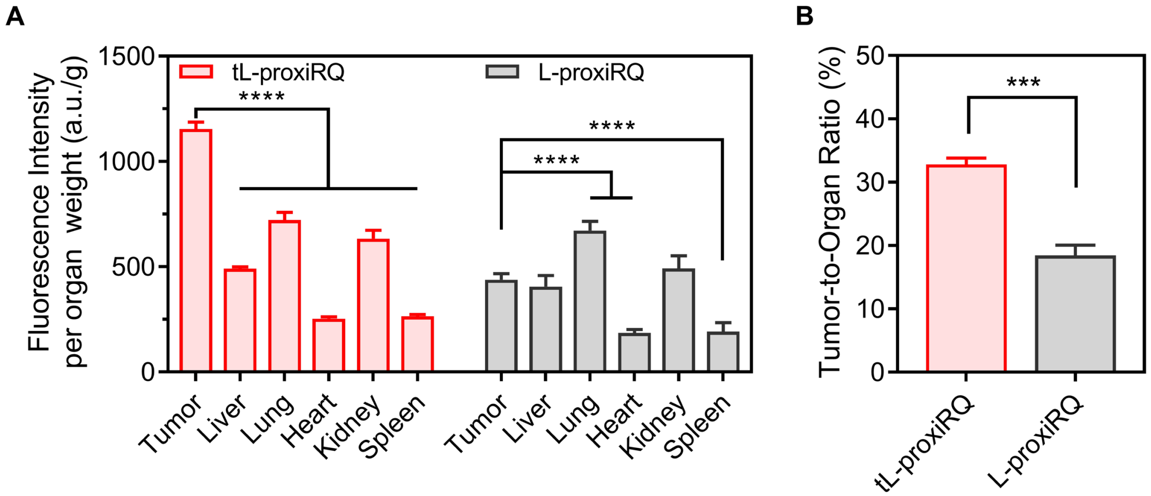

Figure S18. Enhanced tumor targeting efficiency of tL-proxiRQ compared to L-proxiRQ. (A) Quantification of fluorescence intensity normalized to tissue weight in excised tumors and major organs following injection of tL-proxiRQ and L-proxiRQ, for ex vivo biodistribution analysis. $^{****}P < 0.0001$ relative to the tumor group. Values are mean ± s.d. (n = 3). (B) Comparison of tumor-to-organ ratios between tL-proxiRQ and L-proxiRQ. $^{***}P < 0.001$. Values are mean ± s.d. (n = 3).

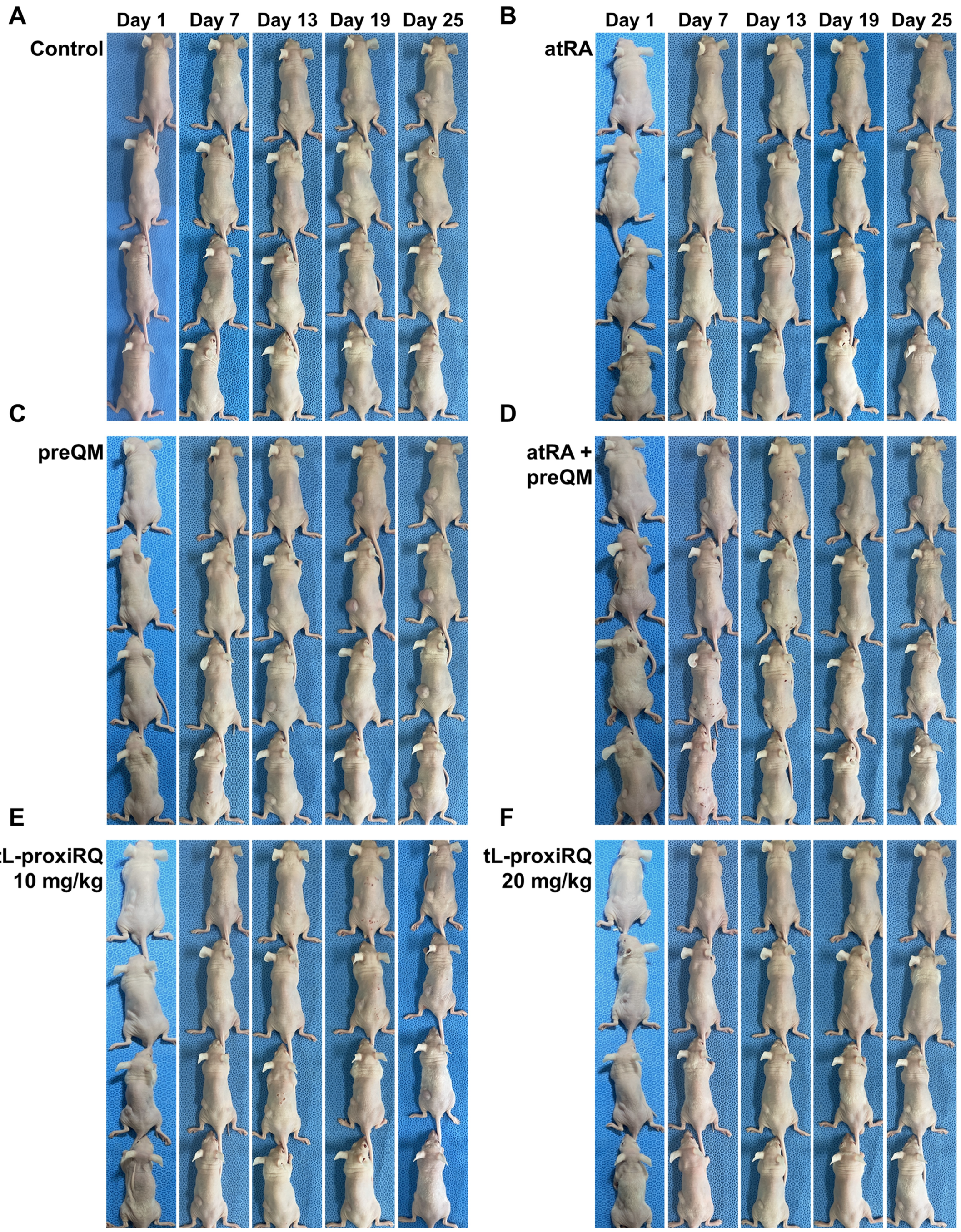

Figure S19. Representative photographs of MCF-7 tumor-bearing mice during treatment. Images were captured on days 1, 7, 13, 19, and 25. Mice were treated with (A) control (saline), (B) atRA, (C) preQM, (D) atRA+preQM, and tL-proxiRQ at doses of (E) 10 mg/kg and (F) 20 mg/kg.

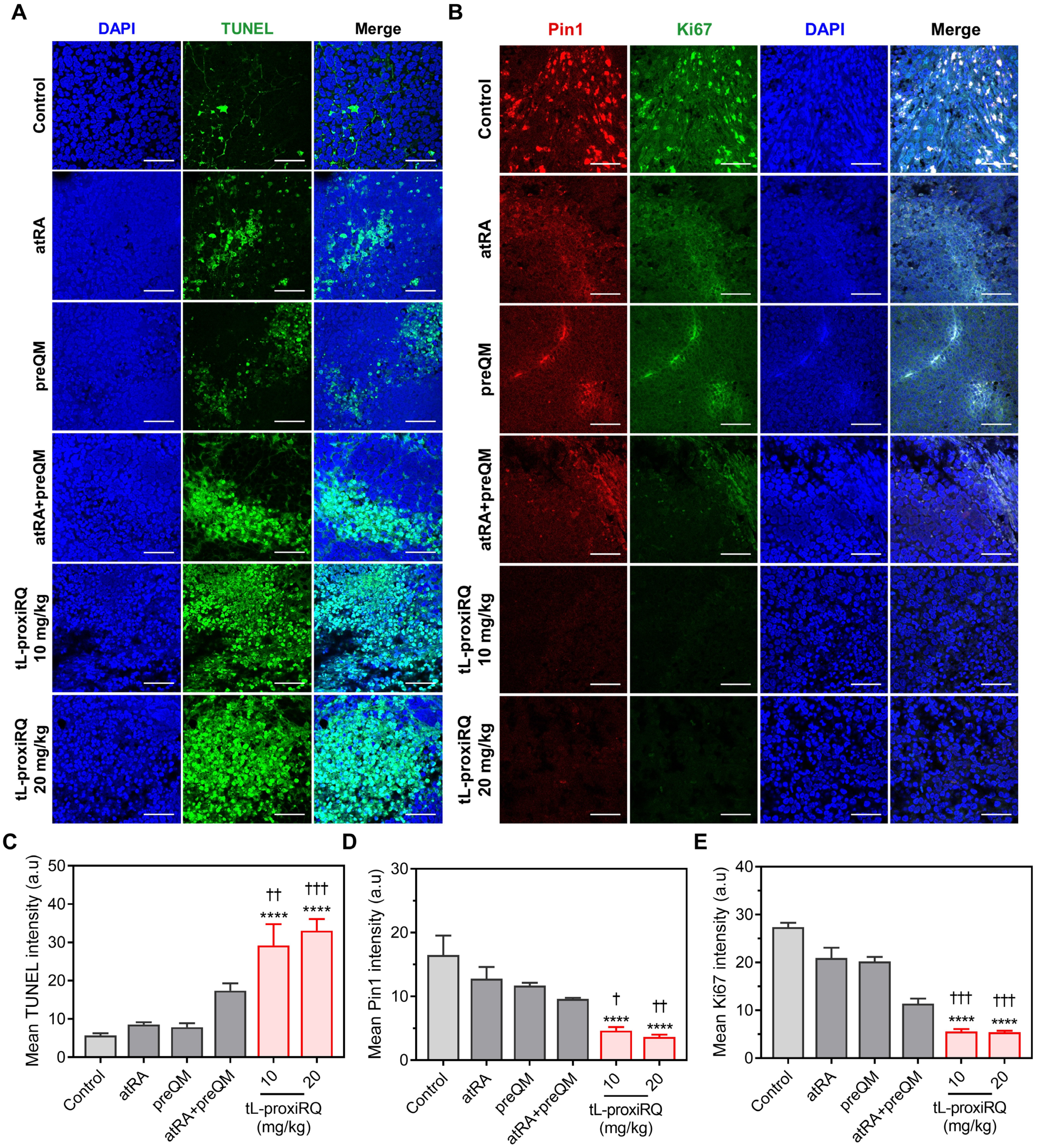

Figure S20. Immunofluorescence analysis of tumor sections after treatment. Representative tumor sections stained for (A) TUNEL, and (B) Pin1 and Ki67. Scale bar is 50 µm. Quantitative analysis of fluorescence intensity for (C) TUNEL, (D) Pin1, and (E) Ki67 using ImageJ software. $^{****}P < 0.0001$ relative to the control. $^{\dagger}P < 0.05$, $^{\dagger\dagger}P < 0.01$, $^{\dagger\dagger\dagger}P < 0.001$ relative to atRA+preQM. Values are mean ± s.d. (n = 4).

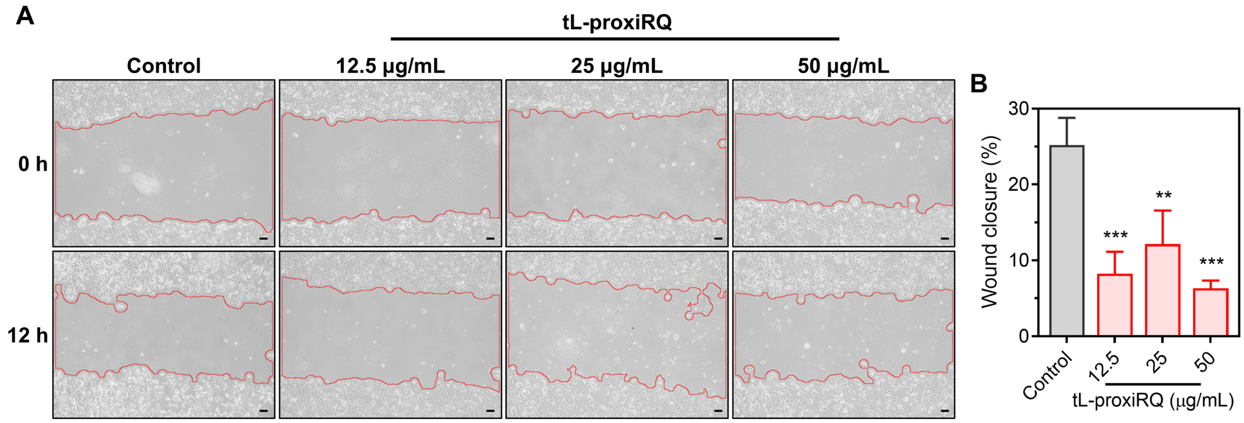

Figure S21. Wound healing assay evaluating the aniti-migratory effect of tL-proxiRQ. (A) Representative images of the MCF-7 cell scratch assay at 0 h and 12 h after treatment. The red lines indicate the migration edges of the cells. Scale bar is 100 μm. (B) Quantitative analysis of the percentage of wound closure. The wound area was measured using ImageJ software, and the percentage of wound closure was calculated relative to the initial wound area at 0 h. $^{**}P < 0.01$, $^{***}P < 0.001$ relative to the control. Values are mean ± s.d. (n = 3).

	Pearson's Coefficient	Spearman's Rank Coefficient	Area Overlap (%)
**0.5 h**	0.701	0.791	71.4
**1 h**	0.821	0.852	70.1
**3 h**	0.826	0.885	64.3
**6 h**	0.824	0.592	10.3

Table S1. Co-localization analysis of cellular uptake and endosomal escape corresponding to Figure 4A. Co-localization analysis was performed on confocal microscopy images using ImageJ software, and the degree of co-localization is expressed as Pearson's coefficient, Spearman's rank coefficient, and area overlap (%).

Human primer			
**No.**	**Name**	**Forward (Sense)**	**Reverse (Antisense)**
1	NFE2L2	CAC ATC CAG TCA GAA ACC AGT GG	GGA ATG TCT GCG CCA AAA GCT G
2	c-Fos	GCC TCT ACT ACC ACT CAC C	AGA TGG CAG TGA CCG TGG GAA
3	c-Jun	CCT TGA AAG CTC AGA ACT CGG AG	TGC TGC GTT AGC ATG AGT TGG C
4	HO-1	CTC AAC ATC CAG CTC TTT GAG	AAT CTT GCA CTT TGT TGC TGG C
5	TBP	CCG AAA CGC CGA ATA TAA TCC	AAA TCA GTG CCG TGG TTC GT

Table S2. Primer sequences used for the qPCR analysis.

Antibodies					
**No.**	**Protein**	**Type of antibody**	**Cat.**	**Dilution**	**Supplier**
1	Pin1	Rabbit polyclonal	3722	1/1000	Cell signalling Technology
2	Cyclin D1	Rabbit polyclonal	2922	1/1000	Cell signalling Technology
3	MMP-9	Rabbit polyclonal	sc-10737	1/1000	Santa Cruz
4	BCL-2	Rabbit monoclonal	3498	1/1000	Cell signalling Technology
5	BAX	Rabbit polyclonal	2772	1/1000	Cell signalling Technology
6	p53	Rabbit polyclonal	9282	1/1000	Cell signalling Technology
7	Caspase-7	Rabbit polyclonal	9492	1/1000	Cell signalling Technology
8	PARP	Rabbit monoclonal	9532	1/1000	Cell signalling Technology
9	β-actin	Mouse monoclonal	sc-47778	1/1000	Santa Cruz
10	GAPDH	Mouse monoclonal	sc-47724	1/1000	Santa Cruz
11	HRP	Anti-rabbit IgG	7074	1/2000	Cell signalling Technology
12	HRP	Anti-mouse IgG	7076	1/2000	Cell signalling Technology

Table S3. List of antibodies used for Western blot analysis.
